# Physiological versus time based cord clamping in very preterm infants (ABC3): a parallel-group, multicentre, randomised, controlled superiority trial

**DOI:** 10.1016/j.lanepe.2024.101146

**Published:** 2024-12-04

**Authors:** Ronny Knol, Emma Brouwer, Thomas van den Akker, Philip L.J. DeKoninck, Wes Onland, Marijn J. Vermeulen, Willem P. de Boode, Anton H. van Kaam, Enrico Lopriore, Irwin K.M. Reiss, G. Jeroen Hutten, Sandra A. Prins, Estelle E.M. Mulder, Esther J. d’Haens, Christian V. Hulzebos, Helene A. Bouma, Sam J. van Sambeeck, Hendrik J. Niemarkt, Mayke E. van der Putten, Tinta Lebon, Inge A. Zonnenberg, Debbie H. Nuytemans, Sten P. Willemsen, Graeme R. Polglase, Sylke J. Steggerda, Stuart B. Hooper, Arjan B. te Pas

**Affiliations:** a Division of Neonatology, Department of Neonatal and Pediatric Intensive Care, Erasmus MC University Medical Center Rotterdam, Rotterdam, the Netherlands; b Division of Neonatology, Department of Pediatrics, Leiden University Medical Center, Leiden, the Netherlands; c Department of Obstetrics, Leiden University Medical Center, Leiden, the Netherlands; d Athena Institute, VU University, Amsterdam, the Netherlands; e Division of Obstetrics and Fetal Medicine, Department of Obstetrics and Gynecology, Erasmus MC University Medical Center Rotterdam, Rotterdam, the Netherlands; f The Ritchie Centre, Hudson Institute of Medical Research, Clayton, Victoria, Australia; g Department of Neonatology, Emma Children’s Hospital, Amsterdam UMC, University of Amsterdam and Vrije Universiteit Amsterdam, the Netherlands; h Amsterdam Reproduction & Development, Amsterdam, the Netherlands; i Division of Neonatology, Department of Pediatrics, Radboud University Medical Center, Radboud Institute for Health Sciences, Amalia Children’s Hospital, Nijmegen, the Netherlands; j Department of Neonatology, Isala Women and Children's Hospital, Zwolle, the Netherlands; k Department of Pediatrics, Beatrix Children’s Hospital, University Medical Center Groningen, Groningen, the Netherlands; l Department of Pediatrics, Maxima Medical Center, Veldhoven, the Netherlands; m Department of Pediatrics, Maastricht University Medical Center, Maastricht, the Netherlands; n Department of Neonatology, Wilhelmina Children’s Hospital, University Medical Center Utrecht, Utrecht, the Netherlands; o Department of Biostatistics, Erasmus MC University Medical Center Rotterdam, Rotterdam, the Netherlands; p Department of Paediatrics, Monash University, Clayton, Victoria, Australia; q Department of Obstetrics and Gynaecology, Monash University, Clayton, Victoria, Australia

**Keywords:** Preterm infants, Cord clamping, Newborn resuscitation

## Abstract

**Background:**

Physiological-based cord clamping (PBCC) in preterm infants is beneficial for cardiovascular transition at birth and may optimize placental transfusion. Whether PBCC can improve clinical outcomes is unknown. The aim of the Aeration, Breathing, Clamping (ABC3) trial was to test whether PBCC results in improved intact survival in very preterm infants.

**Methods:**

The ABC3 trial was a parallel-group, multicentre, randomised, controlled superiority clinical trial conducted in all Dutch tertiary referral centers for perinatal care involving infants born before 30 weeks of gestation. Infants were randomised to either PBCC or time-based delayed cord clamping (TBCC), stratified by gestational age and treatment center. Infants receiving PBCC were stabilised with umbilical cord intact, which was clamped after reaching cardiorespiratory stability (heart rate >100 bpm and SpO2 >85% while supplemental oxygen <40%). In TBCC the cord was clamped after 30–60 s. The primary outcome was survival without major cerebral injury and/or necrotizing enterocolitis. The primary and key secondary analyses were done in both the intention-to-treat and per-protocol populations. The trial was registered with ClinicalTrials.gov (NCT03808051).

**Findings:**

From January 25, 2019, through October 2, 2022, 669 infants were randomised (median gestational age 27^+5^ weeks (IQR 26^+2^–28^+6^)) and included in the intention-to-treat population. Intact survival occurred in 241 of 339 infants (71.1%) after PBCC, compared with 223 of 330 (67.6%) after TBCC (odds ratio 1.18, 95% CI 0.84–1.66; absolute risk difference 3.1 %points, 95% CI −11.0 to 15.8, p = 0.33). Pre-specified subgroup analysis showed 69.9% intact survival in male infants after PBCC, compared with 61.8% after TBCC (odds ratio 2.32, 95% CI 1.42–3.78, p for interaction 0.026). Secondary outcomes showed fewer red blood cell transfusions after PBCC (rate ratio 0.83, 95% CI 0.75–0.92, p = 0.0003), lower incidence of late-onset sepsis (27.4% versus 33.3%, odds ratio 0.77, 95% CI 0.62–0.95, p = 0.013) and lower admission temperature (36.3 °C versus 36.7 °C, mean difference −0.5, 95% CI −0.8 to −0.3, p < 0.0001). Parents were less anxious (Likert scale 1.52 (SD 0.97) versus 2.23 (SD 1.35); p < 0.001) and more content (Likert scale 4.74 (SD 0.75) versus 4.49 (SD 0.97); p < 0.001) after PBCC.

**Interpretation:**

PBCC in very preterm infants did not increase survival without major cerebral injury or necrotizing enterocolitis compared to TBCC in the entire cohort. A possible beneficial effect in male infants requires confirmation from other trials. PBCC was safe to perform and parents reported more contentment and less anxiety.

**Funding:**

The Netherlands Organization for Health Research and Development.

**Research in context**

**Evidence before this study**

Delayed cord clamping has shown to reduce mortality in preterm infants, so delayed clamping for 30–60 s is currently recommended for preterm infants not needing resuscitation. Delaying clamping until lung aeration has been established (physiological-based cord clamping) has shown hemodynamic benefits in experimental settings. For this, stabilisation with intact umbilical cord is needed, and whether this is beneficial in preterm infants was unknown. We searched MEDLINE and the Cochrane Database of Systematic Reviews for studies published in English between Jan 1, 1990 and Dec 31, 2018 reporting randomized, clinical trials and cohort studies relevant to our study, with the search terms “preterm infants”, “physiological based cord clamping”, “delayed cord clamping”, “survival”, and “mortality”. Although we found no clinical studies on physiological-based cord clamping, three trials and 2 observational studies on intact cord resuscitation in preterm infants were published. All studies confirmed feasibility and safety of the approach, but none was powered to show differences in clinical outcomes.

**Added value of this study**

While our primary outcome is not different between the two groups of very preterm infants, clinical improvement in pre-specified subsets of infants and better results in important secondary outcomes after physiological-based cord clamping are important findings in our trial. In addition, our results show that physiological-based cord clamping in very preterm infants is feasible and safe to perform.

**Implications of all the available evidence**

The latest systematic review and network meta-analysis with individual participant data in 2023 concluded that the approach of long deferral of cord clamping (≥120 s) decreases all-cause mortality in preterm infants and has the highest probability of being the best cord clamping approach in preterm infants. The results of our large trial on physiological-based cord clamping provides data to update meta-analyses on optimal cord clamping approaches in preterm infants, which will inform international guidelines.

## Introduction

Systematic reviews have shown that preterm infants benefit from delayed cord clamping (DCC) resulting in fewer blood transfusions and decreased mortality.^1^, ^2^, ^3^ Current international guidelines and consensus statements recommend DCC for 30–60 s for all preterm infants not needing immediate resuscitation.^4^, ^5^, ^6^ A recent network meta-analysis, utilizing individual patient data, demonstrated that the effect on increased survival in preterm infants was strongest, when clamping was delayed for longer than 120 s, compared to other approaches.^7^

Clinically, the current DCC approach is based on a fixed duration that assumes placental transfusion to occur within a set time frame.^4^^,^^5^^,^^8^ Studies in preterm lambs have demonstrated that clamping the cord after the initiation of ventilation maintains cardiac output and prevents large fluctuations in systemic and cerebral blood pressures and flows.^9^^,^^10^ Poor respiratory gas exchange and a loss in cardiac output at birth, as indicated by prolonged hypoxia and bradycardia, are associated with increased risk of intraventricular hemorrhage (IVH) and death.^11^ The strategy of clamping the cord after establishing lung aeration and cardiopulmonary stabilisation, is called physiological-based cord clamping (PBCC).^12^^,^^13^

Previous clinical studies in preterm infants investigating time-based cord clamping (TBCC) with application of respiratory support prior to cord clamping, have shown the feasibility of this approach.^14^, ^15^, ^16^, ^17^ However, these studies were underpowered to demonstrate a difference in clinical outcomes. A recent larger trial was not able to show a decrease in IVH or mortality after TBCC of 2 min with respiratory support on the cord, but this approach did not take into account the variation in time needed to aerate the lungs between infants, which may not have been completed before cord clamping.^18^

We recently demonstrated feasibility and effectiveness of performing PBCC in preterm infants.^19^^,^^20^ The multicentre randomised ABC3 (Aeration, Breathing, Clamping 3) trial in very preterm infants aimed to test the hypothesis that, performing PBCC improves survival without severe cerebral injury and/or necrotizing enterocolitis (NEC).^21^

## Methods

### Study design

This multicentre trial was a parallel group, superiority randomised controlled clinical trial. The trial protocol and the statistical analysis plan were published previously.^21^^,^^22^ Assessment of eligibility and recruitment of patients occurred in all nine Dutch tertiary referral centers for perinatal care. The trial was approved by the Institutional Review Board (IRB) at each center and was conducted according to the principles of the Declaration of Helsinki and in accordance with the Dutch law (Medical Research Involving Human Subjects Act). An independent external Data safety and Monitoring Committee (DMC) assessed and reviewed patient safety and trial conduct.

### Participants

Eligible patients were very preterm infants born before 30 weeks of gestation. Antenatal parental informed consent was necessary for all participants in the trial. Exclusion criteria were significant congenital malformations; signs of acute placental abruption; placenta previa or invasive placentation (accreta/percreta); birth by emergency caesarean section (ordered to be executed within 15 min); monochorionic twin gestation with signs of twin-to-twin transfusion syndrome or twin anemia polycythemia syndrome not treated with fetoscopic laser surgery; multiple pregnancy >2 (triplets or higher order); or a documented decision to give palliative neonatal care. Maternal general anesthesia was an exclusion criterium at the start of the trial, but inclusion was allowed after amendment of the protocol and approval by the IRB. During the COVID19-pandemic inclusion was temporarily halted in some participating centers following hospital regulations.

### Randomisation and masking

Infants were 1:1 randomised to either PBCC or TBCC. Allocation was stratified by gestational age (<27^+0^ and ≥27^+0^ weeks) and treatment center using random permutated block (4–8) sizes. Concealment of allocation was ensured by using the randomisation process of Castor Electronic Data Capture (Amsterdam, The Netherlands, www.castoredc.com). Blinding during the intervention was not possible. Independent assessors, all neonatologists, who verified the primary outcome were blinded for treatment allocation.

In case of twin vaginal birth, both infants were randomised to the same group. In case of caesarean section for twins, it was deemed technically not possible to perform PBCC in both infants. The first infant always received standard delayed cord clamping without randomisation and the second infant was randomised to either PBCC or TBCC.

### Procedures

#### Interventional treatment (PBCC)

A specifically designed trolley (Concord Neonatal B.V., Leiden, The Netherlands) was used to perform PBCC in this trial. The trolley contains similar equipment as a regular resuscitation table. Immediately after birth, the infant was placed on the trolley, respiratory support was commenced applying CPAP and PPV when necessary via facemask, and temperature was managed using a plastic wrap and radiant heater. The umbilical cord was clamped when the infant was stabilised, defined as having a heart rate >100 bpm and SpO_2_ >85% while using <40% supplemental oxygen. The minimum time of cord clamping was 3 min and maximum time 10 min. With the exception of the PBCC procedure, the infants were treated according to current local guidelines, based on international Newborn Life Support resuscitation guidelines. Uterotonic drugs were administered immediately after cord clamping, followed by active management of placental delivery. Excessive maternal blood loss was a predefined reason for earlier cord clamping.

#### Standard treatment (TBCC)

Infants randomised to the control group were stabilised according to standard management. Cord clamping was time-based and performed preferably after 30–60 s, depending on the clinical condition of the infant according to the national delayed cord clamping protocol. The infant was then moved to the standard resuscitation table for cardiopulmonary stabilisation. Respiratory support was commenced applying CPAP and PPV when necessary via facemask, and temperature was managed using a plastic wrap and radiant heater. Infants were treated according to current local guidelines, based on international Newborn Life Support resuscitation guidelines. Uterotonic drugs were administered immediately after cord clamping, followed by active management of placental delivery.

### Outcomes

The primary outcome was the dichotomous outcome of intact survival at Neonatal Intensive Care Unit (NICU) discharge, defined as survival without major cerebral injury (IVH ≥ grade 2 and/or periventricular venous infarction and/or periventricular leukomalacia (PVL) ≥ grade 2) and/or NEC ≥ stage 2. The time frame of observation was from the date of randomisation until the date of death or the date of NICU discharge. Cerebral injury was assessed by ultrasonography performed at pre-defined time points. For the grading of IVH and PVL the definitions of Volpe and de Vries were used, respectively.^23^^,^^24^ NEC was diagnosed according to modified Bell’s staging criteria (Supplementary Table S1).^25^ Each component of the primary outcome was re-assessed by an independent researcher blinded for treatment allocation.

Demographic details and patient characteristics were extracted from medical files. Secondary outcomes were collected during NICU stay and are listed in the statistical analysis plan (Supplementary Table S1). Short-term parental reported outcomes were collected using questionnaires on parental perception of the approach during birth and perinatal stabilisation. The parental questionnaires were sent within one week after birth.

Three pre-specified safety parameters were included as Serious Adverse Events: severe hypothermia at NICU admission (defined by World Health Organization as temperature <32 °C), severe maternal post-partum hemorrhage (defined as estimated blood loss >1000 mL), and rupture of the umbilical cord.

### Statistical analysis

The sample size was determined to detect an increase of intact survival of 10% (from 72% to 82%) with 80% power and test size (alpha) of 5%. The required sample size was 330 randomised participants in each arm. The first-born twins from an anticipated caesarean section were not included in any comparative analysis.^22^

The primary analysis for this study was done on intention-to-treat basis. Per-protocol analysis was performed excluding infants who did not meet the inclusion criteria, or who did not start with the intended strategy for any reason. Software used is R version 3.5.0 for all analyses.

To compare the difference in primary outcome between the two arms, a logistic regression model was estimated using generalized estimating equations with an exchangeable working correlation matrix and non-robust standard errors, to account for the potential correlation in the outcome between siblings and infants within the same center. The response of this model was intact survival at NICU discharge and the covariates were the treatment arm and gestational age. The marginal absolute risk difference (ARD) and bootstrapped confidence intervals were calculated using marginal standardization. A significance level of 5% was used for all tests. No multiplicity adjustment was used.^22^

Secondary outcomes similarly were analyzed using generalized estimating equations. The analyses for the parental reported outcomes were measured on a five-point scale and considered to be continuous. Preplanned subgroup analysis was done based on gestational age (<27^+0^ weeks and ≥27^+0^ weeks), mode of birth and sex of the infant. Additionally, a preplanned exploratory analysis was done on the learning curve of this new intervention. We estimated the effect of the number of previously performed PBCC procedures in a center on the odds ratio of intact survival of the intervention. The DMC performed two interim analyses on safety outcomes after 25% and 50% of the total required infants completed their primary outcome and found no reason to stop the trial early.^22^ The trial was registered with ClinicalTrials.gov (NCT03808051).

### Role of the funding source

The funder of the study had no role in study design, data collection, data analysis, data interpretation, or writing of the report.

## Results

**Fig. 1:**
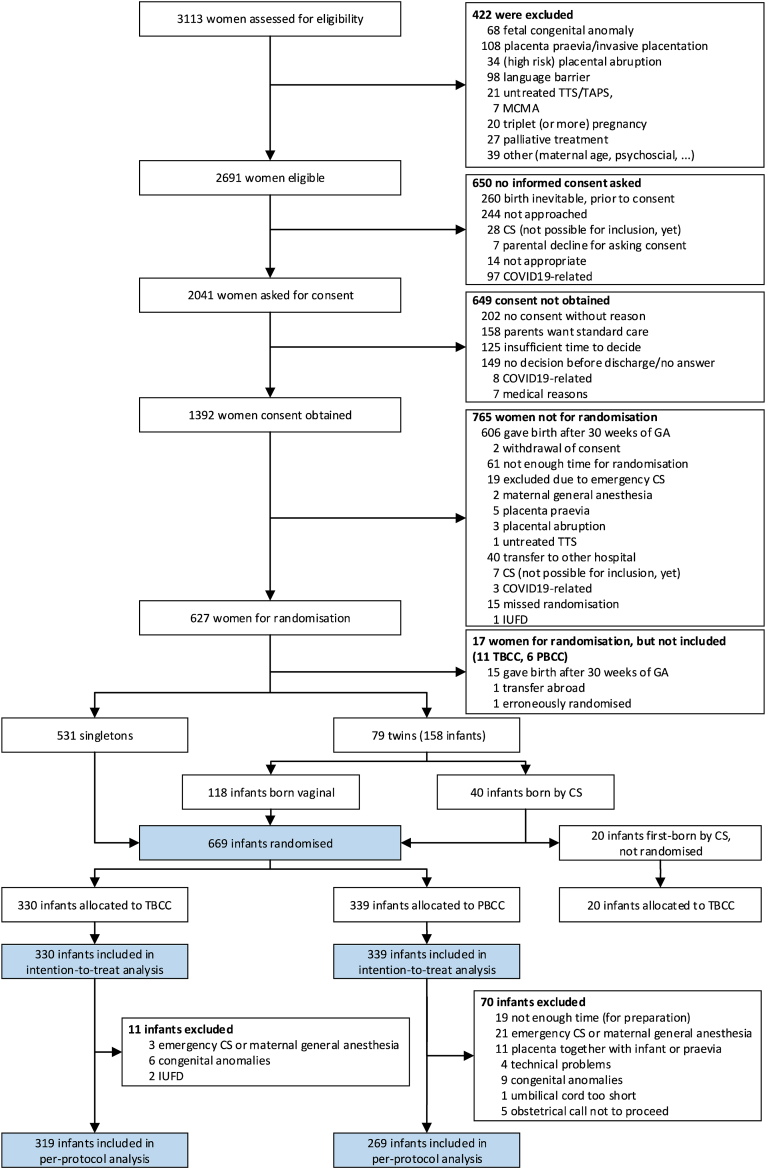
Flow chart of assessment for Eligibility, Consent, Randomization, and Inclusion in the Analysis. TTS = twin transfusion syndrome. TAPS = twin anemia polycythemia syndrome. MCMA = mono-chorionic mono-amniotic twin. CS = caesarean section. COVID19 = Coronavirus disease 2019. GA = gestational age. IUFD = intra uterine fetal demise. TBCC = time-based cord clamping. PBCC = physiological-based cord clamping.

From January 25, 2019, through October 2, 2022, 3113 women at risk for giving birth before 30 weeks of gestation were assessed for eligibility and 1392 women consented for the trial (Fig. 1). Infants of 627 women were randomised, resulting in 669 infants (339 infants in the PBCC and 330 infants in the TBCC group). Baseline characteristics were balanced between the groups (Table 1). Median gestational age was 27^+5^ weeks in both groups. Almost all infants received at least one dose of antenatal corticosteroids.

**Table 1:** Maternal and infant baseline characteristics.

Characteristic	PBCC (N = 339)	TBCC (N = 330)
Maternal		
Age (years)	31.3 (±4.6)	31.9 (±4.9)
Gravidity	1 (1–3)	1 (1–3)
Complications of pregnancy		
Hypertensive disorder of pregnancy	66/309 (21%)	63/302 (21%)
PPROM	93/309 (30%)	95/302 (31%)
Chorioamnionitis	103/290 (36%)	109/287 (38%)
Single gestation	263 (79%)	261 (79%)
Twin gestation		
Monochorionic	21/76 (28%)	20/69 (29%)
Dichorionic	55/76 (72%)	49/69 (71%)
Prenatal steroids	337 (99%)	329 (100%)
Complete	252 (74%)	226 (68%)
Infant		
Gestational age (weeks)	27^+5^ (26^+2^–28^+5^)	27^+5^ (26^+2^–29^+0^)
Gestational age strata		
<27^+0^ weeks	124 (37%)	124 (38%)
≥27^+0^ weeks	215 (63%)	206 (62%)
Birthweight (grams)	985 (758–1215)	990 (810–1200)
Small for gestational age^a^	84 (25%)	76 (23%)
Sex		
Male	184 (54%)	186 (56%)
Female	155 (46%)	144 (44%)
Mode of birth		
Vaginal	173 (51%)	179 (54%)
Caesarean section	166 (49%)	151 (46%)

Data are mean (±SD), n (%), n/N (%), or median (IQR). PBCC = physiological-based cord clamping. TBCC = time-based cord clamping. PPROM = preterm premature rupture of membranes.

a According to Fenton Growth Chart < P_10_.

Data regarding the composite primary outcome were available complete for all 669 infants. Intact survival occurred in 241 of 339 infants (71.1%) after PBCC, and in 223 of 330 infants (67.6%) after TBCC (odds ratio 1.18, 95% CI 0.84–1.66; absolute risk difference 3.1 percentage points, 95% CI −11.0 to 15.8, p = 0.33) (Table 2). There were no differences in the components of the primary outcome between the groups (Table 2).

**Table 2:** Effect of treatment on primary outcome and its components.

A) Intention-to-treat analysis					
	PBCC (n = 339)	TBCC (n = 330)	Odds ratio (95% CI)	ARD (95% CI)	p value
Composite primary outcome					
Survival without major cerebral injury and/or necrotizing enterocolitis	241 (71.1%)	223 (67.6%)	1.18 (0.84–1.66)	3.1 (−11.0 to 15.8)	0.33
Components of primary outcome					
Infant death	46 (13.6%)	46 (13.9%)	0.99 (0.53–1.84)	−0.2 (−8.8 to 14.1)	0.96
Major cerebral injury	57 (16.8%)	55 (16.7%)	1.03 (0.88–1.21)	−0.4 (−6.0 to 10.9)	0.69
Necrotizing enterocolitis	25 (7.4%)	29 (8.8%)	0.83 (0.53–1.32)	−0.6 (−6.0 to 16.1)	0.43

B) Per-protocol analysis					
	PBCC (n = 269)	TBCC (n = 319)	Odds ratio (95% CI)	ARD (95% CI)	p value
Composite primary outcome					
Survival without major cerebral injury and/or necrotizing enterocolitis	196 (72.8%)	218 (68.3%)	1.20 (0.80–1.80)	3.4 (−14.6 to 15.2)	0.38
Components of primary outcome					
Infant death	32 (11.9%)	43 (13.5%)	0.92 (0.47–1.78)	−0.8 (−8.8 to 13.0)	0.80
Major cerebral injury	42 (15.6%)	52 (16.3%)	0.99 (0.75–1.32)	0.2 (−7.1 to 13.9)	0.93
Necrotizing enterocolitis	20 (7.4%)	29 (9.1%)	0.83 (0.49–1.41)	−1.4 (−7.0 to 20.8)	0.50

C) Subgroup analysis of the composite primary outcome: survival without major cerebral injury and/or necrotizing enterocolitis (Intention-to-treat analysis)					
	PBCC (n = 339)	TBCC (n = 330)	Odds ratio (95% CI)	p value within subgroup^a^	p value for interaction
Gestational age					
< 27^+0^ weeks	65/124 (52.4%)	60/124 (48.4%)	1.15 (0.44–2.97)	0.77	
≥ 27^+0^ weeks	176/215 (81.9%)	163/206 (79.1%)	1.21 (0.52–2.81)	0.66	
					0.96
Sex					
Male	128/184 (69.6%)	115/186 (61.8%)	2.32 (1.42–3.78)	0.001	
Female	113/155 (72.9%)	108/144 (75.0%)	0.54 (0.22–1.39)	0.20	
					0.026
Mode of birth					
Vaginal	119/173 (68.8%)	114/179 (63.7%)	1.18 (0.35–3.96)	0.79	
Caesarean section	122/166 (73.5%)	109/151 (72.2%)	1.17 (0.38–3.62)	0.79	
					0.99

Data are n (%) or n/N (%). CI = confidence interval. PBCC = physiological-based cord clamping. TBCC = time-based cord clamping. ARD = absolute risk difference.

Major cerebral injury was defined as intraventricular hemorrhage ≥ grade 2 and/or periventricular venous infarction and/or periventricular leukomalacia ≥ grade 2; Necrotizing enterocolitis was defined as modified Bell’s stage ≥2.

a p value was not adjusted for multiple comparisons.

PBCC resulted in a mean cord clamping time of 5:47 (SD ± 3:09) minutes, compared with 0:47 (SD ± 0:35) minutes after TBCC (difference 5:00 min, 95% CI 4:30–5:30, p < 0.0001) (Table 3). Time to start respiratory support (difference −0:30 min, 95% CI −0:41 to −0:17, p < 0.001) and time to stabilisation (difference −1:44 min, 95% CI −2:14 to −1:16, p < 0.001) were shorter after PBCC. Respiratory support was given to 666 infants (99.6%) during stabilisation at birth. Hemoglobin level (difference 0.5 g/dl, 95% CI 0.2–0.8 g/dl, p = 0.0052) on the first postnatal day was higher after PBCC, and infants in the PBCC group received fewer red blood cell transfusions (rate ratio 0.83, 95% CI 0.75–0.92, p = 0.0003). After PBCC a lower incidence for late onset sepsis (odds ratio 0.77, 95% CI 0.62–0.95), p = 0.013) was observed. No differences were found for other important secondary clinical outcomes, including respiratory distress syndrome, bronchopulmonary dysplasia and retinopathy op prematurity (Table 3 and Supplementary Table S2).

**Table 3:** Secondary outcome measures (Intention-to-Treat Analysis).

A) Characteristics at birth	PBCC (N = 339)	TBCC (N = 330)	Odds ratio (95% CI)	Difference (95% CI)	p value
Time to:					
Start respiratory support (min:sec)	0:59 (±1:08)	1:28 (±0:45)		−0:30 (−0:41 to −0:17)	<0.0001
Infant is stabilized (min:sec)	6:42 (±3:06)	8:24 (±7:24)		−1:44 (−2:14 to −1:16)	<0.0001
Cord clamping (min:sec)	5:47 (±3:09)	0:47 (±0:35)		5:00 (4:30–5:30)	<0.0001
Respiratory support at birth	338 (99.7%)	328 (99.4%)	1.03 (0.05–19.4)		0.99
Type of support at birth					
Supplemental oxygen	307 (90.6%)	287 (87.0%)	1.44 (0.87–2.38)		0.16
CPAP	324 (95.6%)	318 (96.4%)	0.81 (0.30–2.19)		0.68
PPV	224 (66.1%)	197 (59.7%)	1.45 (1.01–1.80)		0.039
Intubation	29 (8.6%)	30 (9.1%)	0.95 (0.44–2.03)		0.89
Chest compressions	2 (0.6%)	5 (1.5%)	0.38 (0.07–2.09)		0.30
Epinephrine	0	3 (0.9%)	NA		0.12
Maximum FiO_2_	0.71 (±0.26)	0.67 (±0.27)		0.04 (−0.01 to 0.08)	0.10
Apgar score					
at 1 min	6 (4–7)	6 (3–7)		0.16 (−0.27 to 0.59)	0.47
at 5 min	8 (7–9)	8 (7–9)		0.04 (−0.21 to 0.29)	0.77
at 10 min	9 (8–9)	9 (8–9)		0.05 (−0.08 to 0.18)	0.45
Umbilical pH	7.23 (7.15–7.31)	7.29 (7.23–7.35)		−0.06 (−0.07 to −0.04)	<0.0001
Rupture of umbilical cord	0	0	NA		
Admission temperature (⁰C)	36.3 (35.6–36.8)	36.7 (36.2–37.2)		−0.5 (−0.8 to −0.3)	<0.0001
**B) Maternal secondary outcomes**	**PBCC (N = 309)**	**TBCC (N = 302)**	**Odds ratio (95% CI)**	**Difference (95% CI)**	

Maternal blood loss (mL)	300 (200–500)	300 (200–500)		9 (−52 to 70)	0.78
Postpartum hemorrhage > 1000 mL	20 (6.5%)	14 (4.6%)	1.3 (0.51–3.52)		0.56
Surgical site infection CS	3/158 (1.9%)	3/148 (2.0%)	0.9 (0.12–6.9)		0.92
**C) Infant secondary outcomes**	**PBCC (N = 339)**	**TBCC (N = 330)**	**Odds ratio (95% CI)**	**Difference (95% CI)**	

Hemoglobin < 24h (g/dl)	17.1 (15.2–19.2)	16.6 (14.8–18.9)		0.5 (0.2–0.8)	0.0052
Hematocrit < 24h (l/l)	0.50 (0.44–0.56)	0.49 (0.44–0.55)		0.01 (0.00–0.02)	0.0020
Polycythemia (Ht > 0.65)	10 (2.9%)	6 (1.8%)	1.58 (0.56–4.44)		0.38
Respiratory Distress Syndrome	226 (66.7%)	207 (62.7%)	1.20 (0.78–1.85)		0.40
Use of surfactant	202 (59.6%)	176 (53.3%)	1.31 (0.85–2.03)		0.12
Intubation < 72h	110 (33.3%)	98 (29.7%)	1.16 (0.76–1.77)		0.13
Volume expansion < 72h	41 (12.1%)	46 (13.9%)	0.84 (0.48–1.49)		0.80
Inotropes < 72h	33 (9.7%)	28 (8.5%)	1.18 (0.62–2.23)		0.50
PDA requiring therapy	48 (14.2%)	52 (15.8%)	0.89 (0.62–1.28)		0.42
Highest bilirubin (umol/l)	139 (116–169)	137 (113–170)		−1.6 (−6.7 to 3.5)	0.54
Hyperbilirubinemia req. therapy	312 (92.0%)	301 (91.2%)	1.04 (0.73–1.48)		0.83
Early onset sepsis	12 (3.5%)	11 (3.3%)	1.07 (0.70–1.63)		0.77
Late onset sepsis	93 (27.4%)	110 (33.3%)	0.77 (0.62–0.95)		0.013
Number of late onset sepsis (n)	0 (0–1)	0 (0–1)	0.75 (0.63–0.89)^a^		0.0010
NEC ≥ stage 2	25 (7.4%)	29 (8.8%)	0.83 (0.53–1.32)		0.43
Red blood cell transfusion	170 (50.1%)	178 (53.9%)	0.85 (0.66–1.08)		0.15
Number of RBC transfusions (n)	1 (0–2)	1 (0–2)	0.83 (0.75–0.92)^a^		0.0003
Intraventricular hemorrhage	91 (26.8%)	102 (30.9%)	0.80 (0.55–1.15)		0.23
Intraventricular hemorrhage grade					
Grade I or II	68 (20.1%)	81 (24.5%)	0.76 (0.49–1.18)		0.22
Grade III or IV	23 (6.8%)	21 (6.4%)	1.08 (0.73–1.62)		0.69
Post hemorrhagic ventricular dilatation	9 (2.7%)	20 (6.1%)	0.42 (0.32–0.55)		<0.0001
Periventricular leukomalacia ≥ grade 2	6 (1.8%)	3 (0.9%)	1.9 (0.65–5.73)		0.24
Number of oxygen days	16 (2–50)	16 (1–54)		−0.8 (−6.1 to 4.6)	0.78
Moderate or severe BPD	65 (19.2%)	78 (23.6%)	0.86 (0.60–1.23)		0.28
Retinopathy of prematurity ≥ stage 2	50 (14.7%)	50 (15.2%)	0.95 (0.80–1.12)		0.11
Treatment for ROP	20 (5.9%)	25 (7.6%)	0.76 (0.38–1.52)		0.44
Length of hospital stay (days)	81 (±27)	83 (±31)		−1.3 (−4.5 to 1.9)	0.42

Data are mean (±SD), n (%), n/N (%), or median (IQR). CI = confidence interval. PBCC=Physiological Based Cord Clamping. ARD = absolute risk difference. CPAP=Continuous Positive Airway Pressure. PPV=Positive Pressure Ventilation. NA=Not Applicable. FiO2=Fraction of inspired Oxygen. CS=Caesarean Section. PDA=Patent Ductus Arteriosus. NEC=Necrotizing Enterocolitis. RBC=Reb Blood Cell. BPD=Bronchopulmonary Dysplasia.

a Rate Ratio instead of Odds Ratio.

**Fig. 2:**
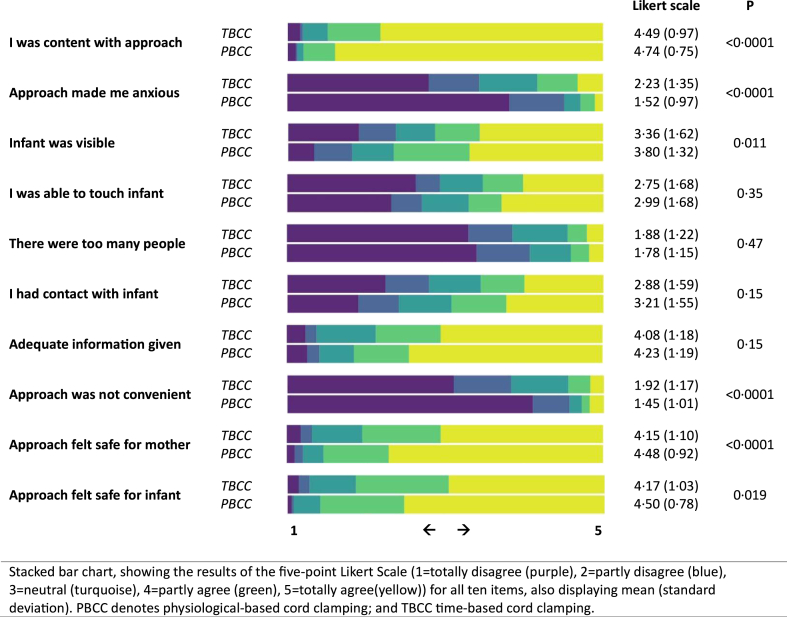
Parental perception and appreciation of the approach during birth and stabilization (Intention-to-Treat Analysis).

Parental response rates to the questionnaire regarding the stabilisation process at birth were 155/339 (46%) in the PBCC group and 107/330 (32%) in the TBCC group. Differences between the groups were observed for contentedness with the approach, degree of anxiety, the visibility of the infant, degree of convenience, and the safety for mother and child, all scoring better in the PBCC group (Fig. 2). Post-hoc non-response analysis showed a higher parental response with higher gestational age and when intact survival was reached (Supplementary Table S5).

For the per-protocol analysis, 70 and 11 infants were excluded respectively, leaving 269 (PBCC) and 319 (TBCC) infants for this analysis. In 61/339 (18.0%) the intended procedure could not be performed in the PBCC group, due to insufficient preparation time (n = 19), emergency caesarian section or maternal general anesthesia (n = 21), placental detachment during birth (n = 11), technical issues (n = 4), too short umbilical cord (n = 1), or obstetric contra-indication (n = 5) (Fig. 1 and Supplementary Table S3). Intact survival occurred in 196 of 269 infants (72.8%) after PBCC and in 218 of 319 infants (68.3%) after TBCC (absolute risk difference 3.4 percentage points, 95% CI −14.6 to 15.2, p = 0.38) (Table 2 and Supplementary Table S4).

Regarding safety, there were no umbilical cord ruptures and no differences between the groups for maternal blood loss, postpartum hemorrhage, polycythemia and hyperbilirubinemia (Table 3). Median infant temperature at admission to the NICU was lower after PBCC (36.3 °C) than after TBCC (36.7 °C) (difference −0.5 °C, 95% CI −0.8 to −0.3 °C, p < 0.0001).

The preplanned intention-to-treat subgroup analysis showed that males had higher intact survival of 69.9% after PBCC versus 61.8% after TBCC (odds ratio 2.32, 95% CI 1.42–3.78, p for interaction 0.026) (Table 1). There were no differences between the groups for the other preplanned subgroups gestational age and mode of birth.

**Fig. 3:**
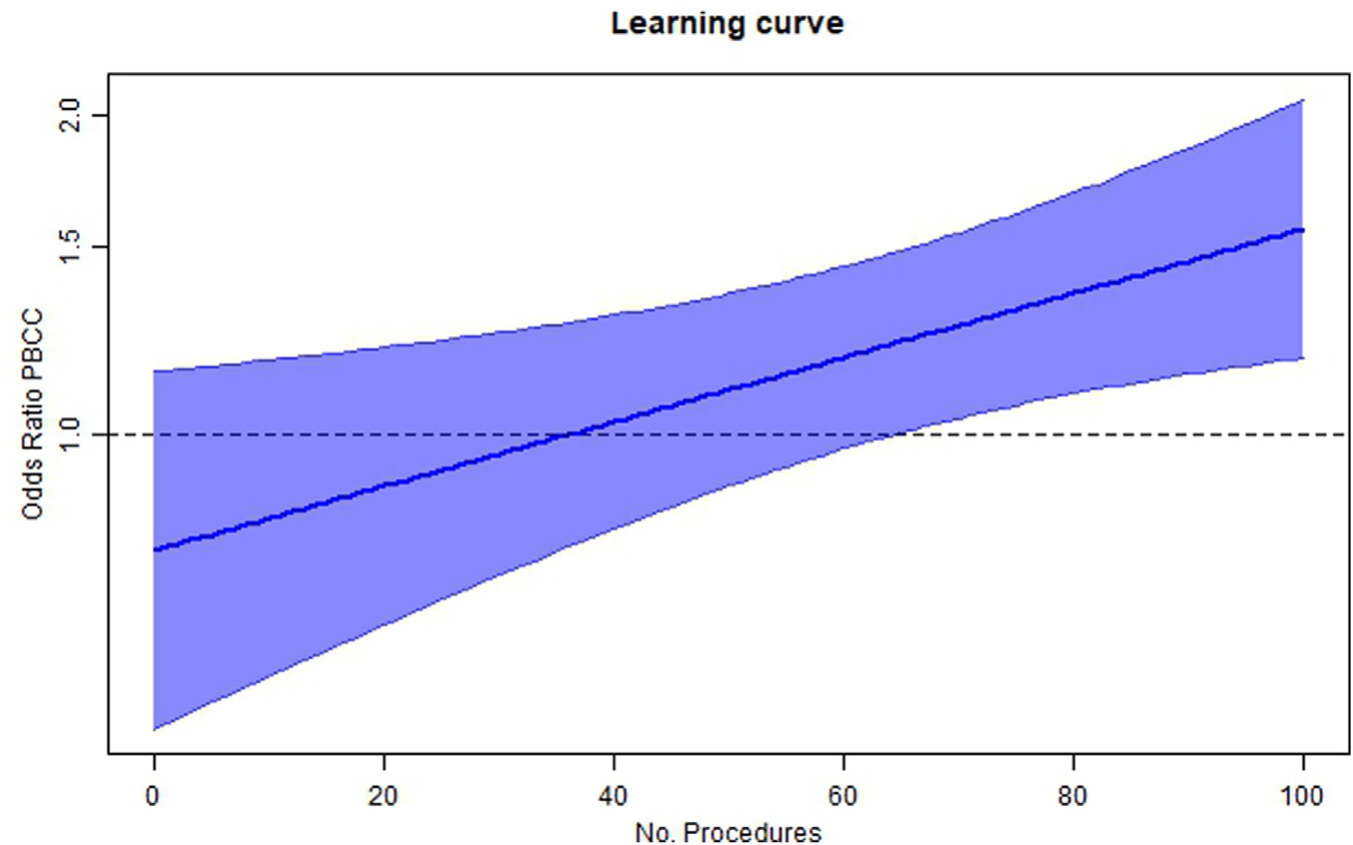
Learning curve: graph showing the estimated effect of the number of previously performed PBCC procedures in a center on the odds ratio (with 95% confidence interval) of intact survival of the intervention (p = 0.010). For each additional procedure the odds of PBCC increases with a factor 1.007 (95% CI 1.002–1.012).

A preplanned exploratory intention-to-treat analysis examined the effect that experience with the PBCC procedure had on the primary outcome (Fig. 3), showing increased difference between the groups, when a center had more experience with the PBCC approach (p = 0.010).

## Discussion

In this multicentre randomised clinical trial comparing physiological-based cord clamping with time-based cord clamping of 30–60 s in very preterm infants, we found that PBCC did not increase survival without major cerebral injury and/or NEC in the entire cohort. This does not confirm the conclusions of a recent meta-analysis comparing different clamping strategies, demonstrating longer deferral times had a high probability of being the best cord clamping approach to decrease infant mortality.^7^ Our trial is the first using cord clamping based on the infant’s clinical condition with delayed cord clamping as comparison, which complicates comparing our results with previous studies, all using time-based cord clamping as intervention and immediate cord clamping as comparison.^14^, ^15^, ^16^, ^17^ More importantly, most previous trials excluded infants needing immediate resuscitation and there was no specific intent to aerate the lungs before clamping.^1^, ^2^, ^3^ We performed delayed cord clamping in the control group, which may have led to insufficient power to demonstrate a significant difference in intact survival when compared to PBCC.

In the present trial we were able to include a large cohort of infants, with a marked difference in cord clamping times between groups. Since it was only possible to perform this trial using antenatal informed consent, a selection of including less sick and well-prepared infants may have influenced the results of this trial, and may limit generalizability.^26^ The experimental physiological evidence suggests that PBCC may have a greater benefit in more unstable preterm infants.^12^ Interestingly, we did notice a significant increase in intact survival in male infants receiving PBCC. Additional analysis showed that the component of major cerebral injury was the main driver of this increase in intact survival (OR 0.34; 95% CI 0.14–0.86). This finding needs to be interpreted with caution, as this is a subgroup analysis, and requires confirmation from other trials. It is previously described that male infants have more difficulty going through transition at birth, need resuscitation more often and have worse outcomes when born prematurely.^27^^,^^28^ Our findings are in line with the recent VentFirst trial, in which preterm infants in the interventional arm received TBCC of 2 min, while respiratory support was provided before clamping.^18^ No difference was observed in the combined primary outcome IVH by age 7–10 days or death before day 7.

Prior to the start of our trial, most study centers required training on the PBCC approach before implementation. Only two centers had gained experience with the PBCC approach before. In each new center training sessions were performed. The fact that in most centers experience was gained during the trial, may have contributed to the reduced effect size between groups. Our pre-specified analysis evaluating the effect of experience on the primary outcome showed an increase in the chance for intact survival as the number of previous PBCC procedures in a participating center increased, which suggests a learning curve that we might have underestimated. This finding requires further exploration, as it stresses the importance of appropriate and careful training as well as obtaining experience, when considering implementation of new clinical approaches.

Strategies to minimize red blood cell transfusions may improve long-term neurodevelopmental outcome in preterm infants.^29^ In our trial, infants in the PBCC group received fewer red blood cell transfusions. It is uncertain whether the observed higher hemoglobin and hematocrit levels in the PBCC group can explain the decrease in transfusions. It is well known that these levels are not the best indicators for estimating total blood volume.^30^ It is also possible that PBCC infants were more stable during admission, resulting in fewer complications and interventions, and fewer blood samples. This is consistent with the finding that PBCC led to a significant decrease in late onset sepsis. Although the mechanism behind this remains unclear and highly speculative, this effect on late onset sepsis was also observed in a previous trial and was attributed to increased numbers of stem cells from the placental circulation reaching the infant.^31^

We hypothesized that PBCC may reduce the incidence of IVH, which was based on the experimental findings that PBCC mitigates the marked increase in arterial blood pressure and cerebral blood flow induced by immediate cord clamping.^9^^,^^10^ The earliest clinical observations of this hypothesis date back to the 1980’s.^32^ While we found no difference in the incidence and severity of IVH, the incidence of post hemorrhagic ventricular dilatation was significantly decreased in the PBCC cohort. While either outcome may be a chance finding, this warrants further exploration.

We did not observe any differences between the groups in maternal safety parameters, including maternal blood loss. The lower umbilical cord pH in the PBCC group has been described in previous trials and is likely due to placental lactate, which merely reflects the timing of cord clamping and not the condition of the infant at birth.^33^^,^^34^ However, we did observe a significantly reduced temperature in PBCC infants at NICU admission. The measures to prevent temperature loss were similar in both groups, however the location where they received these measures differed. Further post-hoc analyses are needed, but it is possible that laminar air flow over the surgery table led to increased heat loss in the PBCC cohort, although this would not explain increased heat loss in vaginally born infants.

In line with earlier reports, we found greater parental contentment and less anxiety after PBCC.^35^^,^^36^ Our data suggest that PBCC is a valuable and safe approach supporting the current trend towards zero separation between mother and infant at birth. We support further development of the approach in this regard.

Our trial has limitations. We were only able to include infants after antenatal consent, resulting in inclusion of infants that were more prepared and stable. No infants below 24 weeks of gestation were included, limiting the generalizability for this group of extremely preterm infants. It was not possible to perform PBCC in all allocated infants, due to various reasons, which is partly explained by the learning curve required for this approach. Nevertheless, our success percentage was comparable to earlier trials.^14^^,^^15^ The results of the secondary analyses should be interpreted with caution, as no adjustments were made for multiplicity. Lastly, as parental response to the surveys was low, it is possible that a higher response rate would yield different results.

In conclusion, we found that physiological-based cord clamping did not increase survival without major cerebral injury or necrotizing enterocolitis in our entire cohort of very preterm infants compared to time-based cord clamping,. A possible beneficial effect in male infants requires confirmation from other trials. Physiological-based cord clamping was safe to perform and parents were more content with this approach.

## Contributors

RK, EB, TvdA, SBH, and AtP designed and planned the study. RK, EB and AtP were the principal investigators. RK, EB, WPdB, IKMR, GJH, SAP, EEMM, CVH, SJvS, MEvdP, IAZ, and AtP were site specific principal investigators. RK, WO, MJV, DHN, and AtP were responsible for the methods. RK, TvdA, PLJD, WO, MJV, WPdB, AHvK, EL, GJH, and AtP were members of the trial steering committee. All study investigators contributed to trial conduct and data collection. RK, WO, MJV, DHN, SPW, and AtP were responsible for data integrity. RK, WO, MJV, SPW, and AtP accessed and verified the data. RK, MJV, SPW, and AtP conducted the statistical analysis. RK, MJV, and AtP wrote the first draft of the manuscript. All authors read, commented on, and approved the final manuscript. All authors had full access to all the data in the study and had final responsibility for the decision to submit for publication.

## Data sharing statement

Study data from the trial are retained and archived for a minimum of 15 years after study completion as per national regulations. All deidentified data generated and analyzed during the trial are available from the corresponding author upon reasonable request.

## Declaration of interests

The authors declare that they have no competing interests. The equipment used in this trial was either designed and built by Leiden University Medical Center (for 3 participating centers) or purchased from Concord Neonatal B.V. (Leiden, The Netherlands). ABtP, SBH, and AHvK are unpaid members of the Scientific Advisory Board of Concord Neonatal B.V. Authors do not have financial relationship with or support from Concord Neonatal. LUMC is owner of the invention and has a license agreement with Concord neonatal, for which LUMC receives royalties. LUMC has the policy to allocate and divide the amount received among the hospital, department and inventors. AtP is one of the inventors. The company had no role in the design of the study; in the collection, analysis, or interpretation of data; or in the writing of the manuscript. MJV is an unpaid board member of the Dutch neonatal patient and parent society Care4Neo.

## Appendix ASupplementary data

Protocol_ABC3_Trials

Supplementary Tables S1–S5

SAP_ABC3_Trials

C1 Protocol_ABC3_v2.2
