# Supplementary material for: Physiological versus time based cord clamping in very preterm infants (ABC3): a parallel-group, multicentre, randomised, controlled superiority trial
Source: Lancet Reg Health Eur. 2024 Dec 4;48:101146. doi: 10.1016/j.lanepe.2024.101146 (PMC11664066; doi:10.1016/j.lanepe.2024.101146)
Supplement: Supplementary Tables S1–S5 [file mmc2.docx]

**Supplementary Appendix**

This appendix has been provided by the authors to give readers additional information about the work

**Table of contents**

Full list of authors, and highest degree.

Table S1. Outcome parameters with definitions.

Table S2. Secondary outcome measures (Intention-to-Treat Analysis).

Table S3. Maternal and Infant Baseline Characteristics (Per Protocol Analysis).

Table S4. Secondary outcome measures (Per-Protocol Analysis).

Table S5. Post-hoc non-response analysis of parental questionnaires regarding the stabilisation process at birth.

**Full list of authors, and highest degree.**

Ronny Knol, M.D.

Emma Brouwer, M.D., Ph.D.

Prof. Thomas van den Akker, M.D., Ph.D.

Philip L.J. DeKoninck, M.D., Ph.D.

Wes Onland, M.D., Ph.D.

Marijn J. Vermeulen, M.D., Ph.D.

Prof. Willem P. de Boode, M.D., Ph.D.

Prof. Anton H. van Kaam, M.D., Ph.D.

Prof. Enrico Lopriore, M.D., Ph.D.

Prof. Irwin K.M. Reiss, M.D., Ph.D.

G. Jeroen Hutten, M.D., Ph.D.

Sandra A. Prins, M.D., Ph.D.

Estelle E.M. Mulder, M.D.

Esther J. d’Haens, M.D.

Christian V. Hulzebos, M.D., Ph.D.

Helene A. Bouma

Sam J. van Sambeeck, M.D., Ph.D.

Hendrik J. Niemarkt, M.D., Ph.D.

Mayke E. van der Putten, M.D.

Tinta Lebon

Inge A. Zonnenberg, M.D., Ph.D.

Debbie H. Nuytemans

Sten P. Willemsen, Ph.D.

Prof. Graeme R. Polglase, Ph.D.

Sylke J. Steggerda, M.D., Ph.D.

Prof. Stuart B. Hooper, Ph.D.

Prof. Arjan B. te Pas, M.D., Ph.D.

| Table S1. Outcome parameters with definitions. | |
| --- | --- |
| Outcome parameter | **Definition** |
|  |  |
| *Primary outcome parameters* |  |
| Mortality | Death due to any cause. |
| Intraventricular hemorrhage (IVH) | Graded according to Volpe.(1) |
| Grade I | Germinal matrix hemorrhage with or without IVH less than 10% of ventricular space. |
| Grade II | Intraventricular hemorrhage occupying 10-50% of ventricular space. |
| Grade III | Intraventricular hemorrhage occupying > 50% of ventricle. |
| Grade IV | Any grade with periventricular venous hemorrhagic infarction. |
| Periventricular venous infarction | Periventricular venous hemorrhagic infarction. |
| Periventricular leukomalacia | Grade according to De Vries.(2) |
| Grade 1 | Persisting increased periventricular echogenicity > 7 days. |
| Grade 2 | Localized small periventricular cysts adjacent to the ventricle. |
| Grade 3 | Extensive periventricular cysts, in fronto-parietal and occipital white matter. |
| Grade 4 | Extensive cysts in periventricular, extending into subcortical white matter. |
| Necrotizing enterocolitis (NEC) | Graded according to modified Bell’s staging criteria.(3) |
| Stage 1 | Suspected NEC; Temperature instability, apnea, bradycardia, lethargy, elevated residuals, mild abdominal distention, bright red blood from  rectum, mild ileus. |
| Stage 2 | Definite NEC; Same as Stage 1, plus metabolic acidosis, thrombocytopenia, absent bowel sounds, definite abdominal tenderness,  +/- abdominal cellulitis or right lower quadrant mass, intestinal dilation, ileus, pneumatosis intestinalis, +/- portal vein gas or ascites. |
| Stage 3 | Advanced NEC; Same as Stage 2, plus hypotension, bradycardia, severe  apnea, combined respiratory and metabolic acidosis, disseminated  intravascular coagulation, neutropenia, signs of generalized peritonitis, marked tenderness, and distention of abdomen, +/- definite ascites or  pneumoperitoneum. |
|  |  |
| *Secondary outcome parameters* |  |
| Respiratory Distress Syndrome | Respiratory symptoms requiring respiratory support with or without extra oxygen; and chest x-ray confirming the diagnosis or treatment with surfactant. |
| Pneumothorax | Extra pleural thoracic air, diagnosed by chest x-ray or thoracocentesis. |
| Number of oxygen days | Cumulative number of days, during which extra oxygen was required for more than 12 hours. |
| Bronchopulmonary Dysplasia | Need for supplemental oxygen (FiO2 >0·21) or positive pressure ventilatory support at a postmenstrual age of 36 weeks after at least 28 cumulative days of supplemental oxygen, including an oxygen  reduction test according to Walsh, if indicated.(4) |
| Mild Bronchopulmonary Dysplasia | Treatment with supplemental oxygen (FiO2 >0·21) for ≥28 days and at 36 weeks postmenstrual age FiO2 0·21 or passed oxygen reduction test. |
| Moderate Bronchopulmonary Dysplasia | Treatment with supplemental oxygen (FiO2 >0·21) for ≥28 days and at 36 weeks postmenstrual age FiO2 <0·30 or failed oxygen reduction test. |
| Severe Bronchopulmonary Dysplasia | Treatment with supplemental oxygen (FiO2 >0·21) for ≥28 days and at 36 weeks postmenstrual age FiO2 >0·30 or positive pressure support, either invasive or non-invasive. |
| Early onset sepsis | Positive blood culture < 72 hours of life and need for antibiotics. |
| Late onset sepsis | Positive blood culture > 72 hours of life and need for antibiotics. |
| Meningitis | Sepsis with antibiotic treatment regimen (dosage and duration) for meningitis, irrespective of spinal fluid culture. |
| Focal intestinal perforation | Isolated perforation in a normal-appearing bowel without features of NEC, such as pneumatosis intestinalis or necrosis. |
| Post hemorrhagic ventricular dilatation | Dilatation of the lateral ventricles after IVH, > 2SD according to Levene Index |
| Retinopathy of prematurity | Graded according to international classification.(5) |
|  |  |

1. Volpe JJ. Intraventricular hemorrhage in the premature infant--current concepts. Part II. Ann Neurol. 1989;25(2):109-16.

2. de Vries LS, Eken P, Dubowitz LM. The spectrum of leukomalacia using cranial ultrasound. Behav Brain Res. 1992;49(1):1-6.

3. Walsh MC, Kliegman RM. Necrotizing enterocolitis: treatment based on staging criteria. Pediatr Clin North Am. 1986;33(1):179-201.

4. Walsh MC, Wilson-Costello D, Zadell A, Newman N, Fanaroff A. Safety, reliability, and validity of a physiologic definition of bronchopulmonary dysplasia. J Perinatol. 2003;23(6):451-6.

5. International Committee for the Classification of Retinopathy of P. The International Classification of Retinopathy of Prematurity revisited. Arch Ophthalmol. 2005;123(7):991-9.

| Table S2. Secondary outcome measures (Intention-to-Treat Analysis). | | | | |  |
| --- | --- | --- | --- | --- | --- |
|  |  |  |  |  |  |
| 1. Characteristics at birth | **PBCC**  **(N=339)** | **TBCC**  **(N=330)** | **Odds ratio**  **(95% CI)** | **Difference**  **(95% CI)** | **p value** |
|  |  |  |  |  |  |
| Time to: |  |  |  |  |  |
| Start respiratory support (min:sec) | 0:59 (±1:08) | 1:28 (±0:45) |  | -0:30 (-0:41 to -0:17) | <0·0001 |
| Infant is stabilized (min:sec) | 6:42 (±3:06) | 8:24 (±7:24) |  | -1:44 (-2:14 to -1:16) | <0·0001 |
| Cord clamping (min:sec) | 5:47 (±3:09) | 0:47 (±0:35) |  | 5:00 (4:30 to 5:30) | <0·0001 |
|  |  |  |  |  |  |
| Respiratory support at birth | 338 (99·7%) | 328 (99·4%) | 1·03 (0·05 to 19·4) |  | 0·99 |
| Type of support at birth |  |  |  |  |  |
| Supplemental oxygen | 307 (90·6%) | 287 (87·0%) | 1·44 (0·87 to 2·38) |  | 0·16 |
| CPAP | 324 (95·6%) | 318 (96·4%) | 0·81 (0·30 to 2·19) |  | 0·68 |
| PPV | 224 (66·1%) | 197 (59·7%) | 1·45 (1·01 to 1·80) |  | 0·039 |
| Intubation | 29 (8·6%) | 30 (9·1%) | 0·95 (0·44 to 2·03) |  | 0·89 |
| Chest compressions | 2 (0·6%) | 5 (1·5%) | 0·38 (0·07 to 2·09) |  | 0·30 |
| Epinephrin | 0 | 3 (0·9%) | NA |  | 0·12 |
| Maximum FiO_2_ | 0·71 (±0·26) | 0·67 (±0·27) |  | 0·04 (-0·01 to 0·08) | 0·10 |
| Apgar score |  |  |  |  |  |
| at 1 minute | 6 (4-7) | 6 (3-7) |  | 0·16 (-0·27 to 0·59) | 0·47 |
| at 5 minutes | 8 (7-9) | 8 (7-9) |  | 0·04 (-0·21 to 0·29) | 0·77 |
| at 10 minutes | 9 (8-9) | 9 (8-9) |  | 0·05 (-0·08 to 0·18) | 0·45 |
| Umbilical pH | 7·23 (7·15-7·31) | 7·29 (7·23-7·35) |  | -0·06 (-0·07 to -0·04) | <0·0001 |
| Rupture of umbilical cord | 0 | 0 | NA |  |  |
| Median admission temperature (⁰C) | 36·3 (35·6-36·8) | 36·7 (36·2-37·2) |  | -0·5 (-0·8 to -0·3) | <0·0001 |
|  |  |  |  |  |  |
| 1. Maternal secondary outcomes | **PBCC**  **(N=309)** | **TBCC**  **(N=302)** | **Odds ratio**  **(95% CI)** | **Difference**  **(95% CI)** |  |
|  |  |  |  |  |  |
| Maternal blood loss (mL) | 300 (200-500) | 300 (200-500) |  | 9 (-52 to 70) | 0·78 |
| Postpartum hemorrhage >1000 mL | 20 (6·5%) | 14 (4·6%) | 1·3 (0·51 to 3·52) |  | 0·56 |
| Surgical site infection after CS | 3/158 (1·9%) | 3/148 (2·0%) | 0·9 (0·12 to 6·9) |  | 0·92 |
| Placental weight (grams) | 244 ± 103 | 240 ± 82 |  | 5 (-9 to 19) | 0·46 |
|  |  |  |  |  |  |
| 1. Infant secondary outcomes | **PBCC**  **(N=339)** | **TBCC**  **(N=330)** | **Odds ratio**  **(95% CI)** | **Difference**  **(95% CI)** |  |
|  |  |  |  |  |  |
| Hemoglobin < 24h (g/dl) | 17·1 (15·2-19·2) | 16·6 (14·8-18·9) |  | 0·5 (0·2 to 0·8) | 0·0052 |
| Hematocrit < 24h (l/l) | 0·50 (0·44-0·56) | 0·49 (0·44-0·55) |  | 0·01 (0·00 to 0·02) | 0·0020 |
| Polycythemia (Ht > 0·65) | 10 (2·9%) | 6 (1·8%) | 1·58 (0·56 to 4·44) |  | 0·38 |
| Respiratory Distress Syndrome | 226 (66·7%) | 207 (62·7%) | 1·20 (0·78 to 1·85) |  | 0·40 |
| Use of surfactant | 202 (59·6%) | 176 (53·3%) | 1·31 (0·85 to 2·03) |  | 0·12 |
| Intubation < 72h | 110 (33·3%) | 98 (29·7%) | 1·16 (0·76 to 1·77) |  | 0·13 |
| Pneumothorax | 6 (1·8%) | 7 (2·1%) | 0·82 (0·32 to 2·11) |  | 0·69 |
| Pulmonary hemorrhage | 5 (1·5%) | 8 (2·4%) | 0·60 (0·17 to 2·19) |  | 0·44 |
| Pulmonary interstitial emphysema | 7 (2·1%) | 3 (0·9%) | 2·37 (0·72 to 7·83) |  | 0·16 |
| Number of oxygen days | 16 (2-50) | 16 (1-54) |  | -0·8 (-6·1 to 4·6) | 0·78 |
| Bronchopulmonary Dysplasia | 125 (36·9%) | 132 (40·0%) | 0·86 (0·60 to 1·23)^a^ |  | 0·28 |
| Mild Bronchopulmonary Dysplasia | 60 (17·7%) | 54 (16·4%) |  |  |  |
| Moderate Bronchopulmonary Dysplasia | 8 (2·4%) | 10 (3·0%) |  |  |  |
| Severe Bronchopulmonary Dysplasia | 57 (16·8%) | 68 (20·6%) |  |  |  |
| Volume expansion < 72h | 41 (12·1%) | 46 (13·9%) | 0·84 (0·48 to 1·49) |  | 0·80 |
| Inotropes < 72h | 33 (9·7%) | 28 (8·5%) | 1·18 (0·62 to 2·23) |  | 0·50 |
| PDA requiring therapy | 48 (14·2%) | 52 (15·8%) | 0·89 (0·62 to 1·28) |  | 0·42 |
| Medical treatment | 44/48 (91·7%) | 52/52 (100%) |  |  |  |
| Surgical treatment | 4/48 (8·3%) | 0 |  |  |  |
| Highest bilirubin (umol/l) | 139 (116-169) | 137 (113-170) |  | -1·6 (-6·7 to 3·5) | 0·54 |
| Hyperbilirubinemia req. therapy | 312 (92·0%) | 301 (91·2%) | 1·04 (0·73 to 1·48) |  | 0·83 |
| Phototherapy | 311/312 (99·7%) | 301/301 (100%) |  |  |  |
| Exchange transfusion | 1/312 (0·3%) | 0 |  |  |  |
| Early onset sepsis | 12 (3·5%) | 11 (3·3%) | 1·07 (0·70 to 1·63) |  | 0·77 |
| Late onset sepsis | 93 (27·4%) | 110 (33·3%) | 0·77 (0·62 to 0·95) |  | 0·013 |
| Number of late onset sepsis (n) | 0 (0-1) | 0 (0-1) | 0·75 (0·63 to 0·89)^¶^ |  | 0·0010 |
| Meningitis | 13 (3·8%) | 12 (3·6%) | 1·02 (0·50 to 2·06) |  | 0·96 |
| Necrotizing enterocolitis | 31 (9·1%) | 38 (11·5%) | 0·83 (0·53 to 1·32)^b^ |  | 0·43 |
| Stage 1 | 6 (1·8%) | 9 (2·7%) |  |  |  |
| Stage 2 | 9 (2·7%) | 10 (3·0%) |  |  |  |
| Stage 3 | 16 (4·7%) | 19 (5·8%) |  |  |  |
| Treatment for NEC |  |  |  |  |  |
| Conservative | 18/31 (58·0%) | 18/38 (46·2%) |  |  |  |
| Surgical | 11/31 (35·5%) | 14/38 (35·9%) |  |  |  |
| Too unstable for surgery | 2/31 (6·5%) | 7/38 (17·9%) |  |  |  |
| Focal intestinal perforation | 9 (2·7%) | 7 (2·1%) | 1·27 (0·23 to 7·08) |  | 0·78 |
| Red blood cell transfusion | 170 (50·1%) | 178 (53·9%) | 0·85 (0·66 to 1·08) |  | 0·15 |
| Number of RBC transfusions (n) | 1 (0-2) | 1 (0-2) | 0·83 (0·75 to 0·92)^¶^ |  | 0·0003 |
| Intraventricular hemorrhage | 91 (26·8%) | 102 (30·9%) | 0·80 (0·55 to 1·15) |  | 0·23 |
| Grade I | 36 (10·6%) | 48 (14·5%) |  |  |  |
| Grade II | 32 (9·4%) | 33 (10·0%) |  |  |  |
| Grade III | 9 (2·7%) | 8 (2·4%) |  |  |  |
| Grade IV | 14 (4·1%) | 13 (3·9%) |  |  |  |
| Post hemorrhagic ventricular dilatation | 9 (2·7%) | 20 (6·1%) | 0·42 (0·32 to 0·55) |  | <0·0001 |
| Lumbar punctures | 3/9 (33·3%) | 5/20 (25·0%) |  |  |  |
| Subcutaneous reservoir | 3/9 (33·3%) | 5/20 (25·0%) |  |  |  |
| Permanent internal drainage | 2/9 (22·2%) | 3/20 (15·0%) |  |  |  |
| Periventricular venous infarction | 14 (4·1%) | 13 (3·9%) | 0·94 (0·49 to 1·78) |  | 0·85 |
| Periventricular leukomalacia | 30 (8·8%) | 16 (4·8%) | 1·9 (0·65 to 5·73)^c^ |  | 0·24 |
| Grade 1 | 24 (7·1%) | 13 (3·9%) |  |  |  |
| Grade 2 | 4 (1·2%) | 3 (0·9%) |  |  |  |
| Grade 3 | 1 (0·3%) | 0 |  |  |  |
| Grade 4 | 1 (0·3%) | 0 |  |  |  |
| Cerebellair hemorrhage | 2 (0·6%) | 6 (1·8%) | 0·32 (0·10 to 1·05) |  | 0·061 |
| Seizures | 2 (0·6%) | 4 (1·2%) | 0·48 (0·10 to 2·40) |  | 0·38 |
| Retinopathy of prematurity | 82 (24·2%) | 77 (23·3%) | 0·95 (0·80 to 1·12)^d^ |  | 0·11 |
| Stage 1 | 32 (9·4%) | 27 (8·2%) |  |  |  |
| Stage 2 | 38 (11·2%) | 34 (10·3%) |  |  |  |
| Stage 3 | 12 (3·5%) | 16 (4·8%) |  |  |  |
| Stage 4 | 0 | 0 |  |  |  |
| Stage 5 | 0 | 0 |  |  |  |
| Treatment for ROP | 20 (5·9%) | 25 (7·6%) | 0·76 (0·38 to 1·52) |  | 0·44 |
| Laser therapy | 20 (5·9%) | 25 (7·6%) |  |  |  |
| Intra-ocular anti-VEGF | 1 (0·3%) | 1 (0·3%) |  |  |  |
| Length of NICU stay (days) | 37 (±27) | 35 (±27) |  | 1·9 (-0·6 to 4·3) | 0·13 |
| Length of hospital stay (days) | 81 (±27) | 83 (±31) |  | -1·3 (-4·5 to 1·9) | 0·42 |
| Weight at hospital discharge (grams) | 3078 (±590) | 2876 (±459) |  | -82 (-173 to 7·7) | 0·073 |
| Length at hospital discharge (cm) | 46·7 (±3·8) | 47·6 (±3·6) |  | -0·8 (-1·6 to -0·1) | 0·036 |
| Head circumference hospital discharge (cm) | 34·3 (±2·5) | 34·4 (±2·1) |  | -0·1 (-0·4 to 0·1) | 0·34 |
|  |  |  |  |  |  |
| Data are mean (±SD), n (%), n/N (%), or median (IQR). CI=confidence interval. PBCC=Physiological Based Cord Clamping. TBCC=Time Based Cord Clamping. CPAP=Continuous Positive Airway Pressure. PPV=Positive Pressure Ventilation. NA=Not Applicable. FiO2=Fraction of inspired Oxygen. CS=Caesarean Section. PDA=Patent Ductus Arteriosus. NEC=Necrotizing Enterocolitis. RBC=Reb Blood Cell. ROP=Retinopathy of Prematurity. VEGF=Vascular Endothelial Growth Factor. NICU=Neonatal Intensive Care Unit.  ^a^ Moderate or severe versus mild or no bronchopulmonary dysplasia  ^b^ Stage 2 or 3 versus stage 1 or no necrotizing enterocolitis  ^c^ Grade 2 or more versus grade 1 or no periventricular leukomalacia  ^d^ Stage 2 or more versus stage 1 or no retinopathy of prematurity  ^¶^ Rate Ratio instead of Odds Ratio. | | | | | |

| Table S3. Maternal and Infant Baseline Characteristics (Per Protocol Analysis). | | |
| --- | --- | --- |
|  | | |
| Characteristic | **PBCC**  **(N=269)** | **TBCC**  **(N=319)** |
|  |  |  |
| Maternal |  |  |
| Age (years) | 31·4 (±4·5) | 31·9 (±4·8) |
| Gravidity | 1 (1-3) | 1 (1-3) |
| Complications of pregnancy |  |  |
| Hypertensive disorder of pregnancy | 57/251 (23%) | 63/293 (22%) |
| PPROM | 72/251 (29%) | 91/293 (31%) |
| Chorioamnionitis | 84/236 (36%) | 105/278 (38%) |
| Single gestation | 217 (81%) | 253 (79%) |
| Twin gestation |  |  |
| Monochorionic | 13/52 (25%) | 20/66 (30%) |
| Dichorionic | 39/52 (75%) | 46/66 (70%) |
| Prenatal steroids | 267 (99%) | 318 (100%) |
| Complete | 193 (72%) | 219 (69%) |
|  |  |  |
| Infant |  |  |
| Gestational age (weeks) | 27^+6^ (26^+4^-28^+5^ ) | 27^+5^ (26^+2^-29^+0^) |
| Gestational age strata |  |  |
| < 27+0 weeks | 90 (33%) | 119 (37%) |
| ≥ 27+0 weeks | 179 (67%) | 200 (63%) |
| Birthweight (grams) | 985 (790-1225) | 990 (810-1200) |
| Small for gestational age^¶^ | 67 (25%) | 75 (24%) |
| Sex |  |  |
| Male | 143 (53%) | 177 (55%) |
| Female | 126 (47%) | 142 (45%) |
| Mode of birth |  |  |
| Vaginal | 150 (56%) | 172 (54%) |
| Caesarean section | 119 (44%) | 147 (46%) |
|  |  |  |
| Data are mean (±SD), n (%), n/N (%), or median (IQR). PBCC=physiological-based cord clamping. TBCC=time-based cord clamping. PPROM=preterm premature rupture of membranes.  ^¶^ According to Fenton Growth Chart <P_10_ | | |

| Table S4. Secondary outcome measures (Per-Protocol Analysis). | | | | |
| --- | --- | --- | --- | --- |
|  |  |  |  |  |
| 1. Characteristics at birth | **PBCC**  **(N=269)** | **TBCC**  **(N=319)** | **Odds ratio**  **(95% CI)** | **Difference**  **(95% CI)** |
|  |  |  |  |  |
| Time to: |  |  |  |  |
| Start respiratory support (min:sec) | 0:58 (±1:11) | 1:28 (±0:46) |  | -0:31 (-0:43 to -0:19) |
| Infant is stabilized (min:sec) | 6:30 (±2:48) | 8:30 (±7:30) |  | -1:56 (-2:17 to -1:36) |
| Cord clamping (min:sec) | 6:44 (±2:29) | 0:47 (±0:35) |  | 5:57 (5:25 to 6:29) |
|  |  |  |  |  |
| Respiratory support at birth | 268 (99·6%) | 319 (100%) |  |  |
| Type of support at birth |  |  |  |  |
| Supplemental oxygen | 243 (90·7%) | 281 (88·1%) | 1·28 (0·75 to 2·17) |  |
| CPAP | 258 (96·3%) | 311 (97·5%) | 0·60 (0·19 to 1·83) |  |
| PPV | 176 (65·4%) | 192 (60·2%) | 1·33 (1·01 to 1·74) |  |
| Intubation | 19 (7·1%) | 28 (8·8%) | 0·84 (0·39 to 1·80) |  |
| Chest compressions | 1 (0·4%) | 5 (1·6%) | 0·23 (0·03 to 1·84) |  |
| Epinephrin | 0 | 3 (0·9%) | NA |  |
| Maximum FiO_2_ | 0·70 (±0·26) | 0·67 (±0·28) |  | 0·03 (-0·1 to 0·08) |
| Apgar score |  |  |  |  |
| at 1 minute | 6 (4-7) | 6 (3-7) |  | 0·24 (-0·17 to 0·65) |
| at 5 minutes | 8 (7-9) | 8 (7-9) |  | 0·06 (-0·21 to 0·33) |
| at 10 minutes | 9 (8-9) | 9 (8-9) |  | 0·15 (0·01 to 0·29) |
| Umbilical pH | 7·22 (7·14-7·30) | 7·29 (7·23-7·35) |  | -0·07 (-0·09 to -0·05) |
| Rupture of umbilical cord | 0 | 0 | NA |  |
| Admission temperature (⁰C) | 36·2 (35·5-36·8) | 36·6 (36·2-37·2) |  | -0·6 (-0·9 to -0·3) |
|  |  |  |  |  |
| 1. Maternal secondary outcomes | **PBCC**  **(N=251)** | **TBCC**  **(N=293)** | **Odds ratio**  **(95% CI)** | **Difference**  **(95% CI)** |
|  |  |  |  |  |
| Maternal blood loss (mL) | 300 (200-500) | 300 (200-500) |  | 4 (-66 to 75) |
| Postpartum hemorrhage >1000 mL | 17 (6·8%) | 13 (4·4%) | 1·56 (0·55 to 4·47) |  |
| Surgical site infection after CS | 3/119 (2·5%) | 3/147 (2·0%) | 1·23 (0·16 to 9·20) |  |
| Placental weight (grams) | 237 (±96) | 239 (±81) |  | -4 (-14 to 6) |
|  |  |  |  |  |
| 1. Infant secondary outcomes | **PBCC**  **(N=269)** | **TBCC**  **(N=319)** | **Odds ratio**  **(95% CI)** | **Difference**  **(95% CI)** |
|  |  |  |  |  |
| Hemoglobin < 24h (g/dl) | 17·1 (15·3-19·2) | 16·4 (14·7-18·9) |  | 0·5 (0·2 to 0·8) |
| Hematocrit < 24h (l/l) | 0·51 (0·44-0·56) | 0·49 (0·44-0·54) |  | 0·01 (0·01 to 0·02) |
| Polycythemia (Ht > 0·65) | 10 (4·3%) | 6 (2·2%) | 1·84 (0·70 to 4·8) |  |
| Respiratory Distress Syndrome | 180 (66·9%) | 201 (63·0%) | 1·25 (0·77 to 2·0) |  |
| Use of surfactant | 157 (58·4%) | 170 (53·3%) | 1·31 (0·82 to 2·1) |  |
| Intubation < 72h | 80 (29·7%) | 93 (29·2%) | 1·10 (0·69 to 1·8) |  |
| Pneumothorax | 5 (1·9%) | 6 (1·9%) | 0·97 (0·40 to 2·4) |  |
| Pulmonary hemorrhage | 3 (1·1%) | 8 (2·5%) | 0·45 (0·08 to 2·5) |  |
| Pulmonary interstitial emphysema | 4 (1·5%) | 3 (0·9%) | 1·75 (0·38 to 8·0) |  |
| Number of oxygen days | 14 (1-49) | 16 (1-54) |  | -1·4 (-5·7 to 2·9) |
| Bronchopulmonary Dysplasia | 94 (34·9%) | 127 (39·8%) | 0·83 (0·59 to 1·16)^a^ |  |
| Mild Bronchopulmonary Dysplasia | 41 (15·2%) | 52 (16·3%) |  |  |
| Moderate Bronchopulmonary Dysplasia | 7 (2·6%) | 10 (3·1%) |  |  |
| Severe Bronchopulmonary Dysplasia | 46 (17·1%) | 65 (20·4%) |  |  |
| Volume expansion < 72h | 27 (10·0%) | 46 (14·4%) | 0·68 (0·34 to 1·37) |  |
| Inotropes < 72h | 22 (8·2%) | 27 (8·5%) | 1·03 (0·50 to 2·1) |  |
| PDA requiring therapy | 39 (14·5%) | 51 (16·0%) | 0·83 (0·60 to 1·1) |  |
| Medical treatment | 35/39 (89·7%) | 51/51 (100%) |  |  |
| Surgical treatment | 4/39 (10·3%) | 0 |  |  |
| Highest bilirubin (umol/l) | 140 (115-172) | 137 (113-170) |  | -1·0 (-6·1 to 4·2) |
| Hyperbilirubinemia req. therapy | 249 (92·6%) | 294 (92·2%) | 1·0 (0·61 to 1·8) |  |
| Phototherapy | 249/249 (100%) | 294/294 (100%) |  |  |
| Exchange transfusion | 0 | 0 |  |  |
| Early onset sepsis | 11 (4·1%) | 10 (3·1%) | 1·34 (0·87 to 2·1) |  |
| Late onset sepsis | 73 (27·1%) | 106 (33·2%) | 0·79 (0·65 to 0·95) |  |
| Number of late onset sepsis (n) | 0 (0 -1) | 0 (0 - 1) | 0·76 (0·65 to 0·88)^¶^ |  |
| Meningitis | 10 (3·7%) | 12 (3·8%) | 1·0 (0·40 to 2·5) |  |
| Necrotizing enterocolitis | 23 (8·6%) | 37 (11·6%) | 0·83 (0·49 to 1·41)^b^ |  |
| Stage 1 | 3 (1·1%) | 8 (2·5%) |  |  |
| Stage 2 | 8 (3·0%) | 10 (3·1%) |  |  |
| Stage 3 | 12 (4·5%) | 19 (6·0%) |  |  |
| Treatment for NEC |  |  |  |  |
| Conservative | 13/23 (56·5%) | 17/37 (45·9%) |  |  |
| Surgical | 8/23 (34·8%) | 14/37 (37·8%) |  |  |
| Too unstable for surgery | 2/23 (8·7%) | 7/37 (18·9%) |  |  |
| Focal intestinal perforation | 8 (3·0%) | 7 (2·2%) | 1·4 (0·25 to 8·4) |  |
| Red blood cell transfusion | 129 (48·0%) | 174 (54·5%) | 0·77 (0·70 to 0·85) |  |
| Number of RBC transfusions (n) | 1 (0-1) | 1 (0-2) | 0·76 (0·61 to 0·92)^¶^ |  |
| Intraventricular hemorrhage | 74 (27·5%) | 98 (30·7%) | 0·98 (0·62 to 1·57) |  |
| Grade I | 33 (12·3%) | 47 (14·7%) |  |  |
| Grade II | 24 (8·9%) | 31 (9·7%) |  |  |
| Grade III | 6 (2·2%) | 8 (2·5%) |  |  |
| Grade IV | 11 (4·1%) | 12 (3·8%) |  |  |
| Post hemorrhagic ventricular dilatation | 6 (2·2%) | 20 (6·3%) | 0·35 (0·16 to 0·77) |  |
| Lumbar punctures | 2/6 (33·3%) | 5/20 (25·0%) |  |  |
| Subcutaneous reservoir | 1/6 (16·7%) | 5/20 (25·0%) |  |  |
| Permanent internal drainage | 1/6 (16·7%) | 3/20 (15·0%) |  |  |
| Periventricular venous infarction | 11 (4·1%) | 12 (3·8%) | 1·14 (0·48 to 2·2) |  |
| Periventricular leukomalacia | 23 (8·6%) | 16 (5·0%) | 1·8 (0·76 to 4·3)^c^ |  |
| Grade 1 | 19 (7·1%) | 13 (4·1%) |  |  |
| Grade 2 | 4 (1·5%) | 3 (0·9%) |  |  |
| Grade 3 | 0 | 0 |  |  |
| Grade 4 | 0 | 0 |  |  |
| Cerebellair hemorrhage | 2 (0·7%) | 6 (1·9%) | 0·86 (0·58 to 1·3) |  |
| Seizures | 2 (0·7%) | 2 (0·6%) | 1·26 (0·27 to 5·7) |  |
| Retinopathy of prematurity | 60 (22·3%) | 73 (22·9%) | 0·90 (0·69 to 1·2)^d^ |  |
| Stage 1 | 23 (8·6%) | 26 (8·2%) |  |  |
| Stage 2 | 26 (9·7%) | 32 (10·0%) |  |  |
| Stage 3 | 11 (4·1%) | 15 (4·7%) |  |  |
| Stage 4 | 0 | 0 |  |  |
| Stage 5 | 0 | 0 |  |  |
| Treatment for ROP | 17 (6·3%) | 24 (7·5%) | 0·93 ( 0·43 to 2·0) |  |
| Laser therapy | 17 (6·3%) | 24 (7·5%) |  |  |
| Intra-ocular anti-VEGF | 1 (0·4%) | 1 (0·3%) |  |  |
| Length of NICU stay (days) | 36 (±28) | 35 (±26) |  | 2·2 (0·42 to 4·0) |
| Length of hospital stay (days) | 80 (±24) | 82 (±30) |  | -1·4 (-4·9 to 2·0) |
| Weight at hospital discharge (grams) | 3075 (±605) | 3131 (±654) |  | -45 (-152 to 61) |
| Length at hospital discharge (cm) | 46·6 (±3·7) | 47·5 (±3·6) |  | -0·9 (-1·8 to 0·0) |
| Head circumference at hospital discharge (cm) | 34·2 (±2·5) | 34·4 (±2·1) |  | -0·2 (-0·5 to 0·1) |
|  |  |  |  |  |
| Data are mean (±SD), n (%), n/N (%), or median (IQR). CI=confidence interval. PBCC=Physiological Based Cord Clamping. TBCC=Time Based Cord Clamping. CPAP=Continuous Positive Airway Pressure. PPV=Positive Pressure Ventilation. NA=Not Applicable. FiO2=Fraction of inspired Oxygen. CS=Caesarean Section. PDA=Patent Ductus Arteriosus. NEC=Necrotizing Enterocolitis. RBC=Reb Blood Cell. ROP=Retinopathy of Prematurity. VEGF=Vascular Endothelial Growth Factor. NICU=Neonatal Intensive Care Unit.  ^a^ Moderate or severe versus mild or no bronchopulmonary dysplasia  ^b^ Stage 2 or 3 versus stage 1 or no necrotizing enterocolitis  ^c^ Grade 2 or more versus grade 1 or no periventricular leukomalacia  ^d^ Stage 2 or more versus stage 1 or no retinopathy of prematurity  ^¶^ Rate Ratio instead of Odds Ratio. | | | | |

| Table S5. Post-hoc non-response analysis of parental questionnaires regarding the stabilisation process at birth. | | | | |
| --- | --- | --- | --- | --- |
|  |  | |  | |
|  | **PBCC**  **(N=339)** | | **TBCC**  **(n=330)** | |
|  | **Responders**  **n = 155 (46%)** | **Non-responders**  **n = 184 (54%)** | **Responders**  **n = 107 (32%)** | **Non-responders**  **n = 223 (67%)** |
|  |  |  |  |  |
| Primary outcome: Intact survival | 120/155 (77%) | 121/184 (66%) | 73/107 (68%) | 150/223 (67%) |
| GA < 27 weeks | 49/155 (32%) | 75/184 (41%) | 37/107 (35%) | 87/223 (39%) |
|  |  |  |  |  |
| Data are n/N (%).PBCC=Physiological Based Cord Clamping. TBCC=Time Based Cord Clamping. GA=Gestational age. | | | | |
