# Supplementary material for: Physiological versus time based cord clamping in very preterm infants (ABC3): a parallel-group, multicentre, randomised, controlled superiority trial
Source: Lancet Reg Health Eur. 2024 Dec 4;48:101146. doi: 10.1016/j.lanepe.2024.101146 (PMC11664066; doi:10.1016/j.lanepe.2024.101146)
Supplement: SAP_ABC3_Trials [file mmc3.pdf]

Willemsen *et al. Trials* (2024) 25:164
https://doi.org/10.1186/s13063-024-08014-y

Trials

UPDATE

Open Access

Physiological-based cord clamping in very preterm infants: the Aeration, Breathing, Clamping 3 (ABC3) trial—statistical analysis plan for a multicenter randomized controlled trial

Sten P. Willemsen[1,2], Ronny Knol[1,3*], Emma Brouwer[3], Thomas van den Akker[4,5], Philip L. J. DeKoninck[6,7], Enrico Lopriore[3], Wes Onland[8,9], Willem P. de Boode[10], Anton H. van Kaam[8,9], Debbie H. Nuytemans[8], Irwin K. M. Reiss[1], G. Jeroen Hutten[8,9], Sandra A. Prins[8,9], Estelle E. M. Mulder[11], Christian V. Hulzebos[12], Sam J. van Sambeeck[13], Mayke E. van der Putten[14], Inge A. Zonnenberg[15], Arjan B. te Pas[3] and Marijn J. Vermeulen[1]

Abstract

Background Mortality, cerebral injury, and necrotizing enterocolitis (NEC) are common complications of very preterm birth. An important risk factor for these complications is hemodynamic instability. Pre-clinical studies suggest that the timing of umbilical cord clamping affects hemodynamic stability during transition. Standard care is time-based cord clamping (TBCC), with clamping irrespective of lung aeration. It is unknown whether delaying cord clamping until lung aeration and ventilation have been established (physiological-based cord clamping, PBCC) is more beneficial. This document describes the statistical analyses for the ABC3 trial, which aims to assess the efficacy and safety of PBCC, compared to TBCC.

Methods The ABC3 trial is a multicenter, randomized trial investigating PBCC (intervention) versus TBCC (control) in very preterm infants. The trial is ethically approved. Preterm infants born before 30 weeks of gestation are randomized after parental informed consent.

The primary outcome is intact survival, defined as the composite of survival without major cerebral injury and/or NEC. Secondary short-term outcomes are co-morbidities and adverse events assessed during NICU admission, parental reported outcomes, and long-term neurodevelopmental outcomes assessed at a corrected age of 2 years.

To test the hypothesis that PBCC increases intact survival, a logistic regression model will be estimated using generalized estimating equations (accounting for correlation between siblings and observations in the same center) with treatment and gestational age as predictors. This plan is written and submitted without knowledge of the data.

Discussion The findings of this trial will provide evidence for future clinical guidelines on optimal cord clamping management at birth.

Trial registration ClinicalTrials.gov NCT03808051. Registered on 17 January 2019.

*Correspondence:
Ronny Knol
r.knol@erasmusmc.nl
Full list of author information is available at the end of the article

© The Author(s) 2024. **Open Access** This article is licensed under a Creative Commons Attribution 4.0 International License, which permits use, sharing, adaptation, distribution and reproduction in any medium or format, as long as you give appropriate credit to the original author(s) and the source, provide a link to the Creative Commons licence, and indicate if changes were made. The images or other third party material in this article are included in the article's Creative Commons licence, unless indicated otherwise in a credit line to the material. If material is not included in the article's Creative Commons licence and your intended use is not permitted by statutory regulation or exceeds the permitted use, you will need to obtain permission directly from the copyright holder. To view a copy of this licence, visit http://creativecommons.org/licenses/by/4.0/. The Creative Commons Public Domain Dedication waiver (http://creativecommons.org/publicdomain/zero/1.0/) applies to the data made available in this article, unless otherwise stated in a credit line to the data.

Keywords Preterm infants, Physiological-based cord clamping, Randomized clinical trial, Statistical Analysis Plan, Cord clamping

Background

The purpose of this document is to describe the statistical analyses to be conducted for the Aeration, Breathing, Clamping 3 (ABC3) trial. The details of the study are further described in the trial protocol, published previously [1]. This statistical analysis plan is written and submitted without knowledge of the data.

International guidelines recommend delayed umbilical cord clamping (DCC) up to 1 min in preterm infants, unless the condition of the infant requires immediate resuscitation [2, 3]. Delaying cord clamping until lung aeration and ventilation have been established (physiological-based cord clamping (PBCC)) may allow for a more adequately established pulmonary circulation and may result in a more stable circulatory transition at birth [4, 5]. The hypothesis in the ABC3 trial is that PBCC, compared to time-based cord clamping (TBCC), results in a more stable transition in very preterm infants, leading to improved clinical outcomes.

Methods

Aim of the trial

The ABC3 trial investigates and tests the hypothesis that physiological-based cord clamping (PBCC, intervention) will lead to an increase in intact survival (survival without significant cerebral injury and/or necrotizing enterocolitis (NEC)), when compared to time-based delayed cord clamping (TBCC, standard treatment) in very preterm infants.

Trial design

The ABC3 trial is a parallel-group, multicenter superiority randomized controlled clinical trial, run in The Netherlands, in which infants are randomized between the interventional PBCC group and TBCC [1].

Primary outcome

The primary outcome is the dichotomous outcome of intact survival at NICU discharge, defined as survival without major cerebral injury and/or intestinal injury (i.e., IVH $\geq$ grade 2 and/or PVL $\geq$ grade 2 and/or periventricular venous infarction and/or modified NEC Bell's stage $\geq$ 2). The time frame of observation is from the date of randomization until the date of death or the date of NICU discharge, whichever came first. Each component of the primary outcome will be reviewed by an independent researcher blinded for treatment allocation.

Study population screening

In recruiting centers, all consecutive pregnant women at risk for preterm birth before 30 weeks of gestation are screened to check for eligibility for the trial. If not eligible, no further information is collected. In case of eligibility, either randomization will follow or the reason for no randomization (e.g., no consent or no birth before 30 weeks) will be recorded.

Eligibility

Eligible patients are preterm infants born at < 30 weeks of gestation in one of the participating centers after obtaining parental informed consent. The exclusion criteria are:

- Significant congenital malformations
- Signs of acute placental abruption
- Total placenta previa, anterior placenta previa, or invasive placentation
- Birth by emergency caesarean section
- Twin gestation with signs of twin transfusion syndrome or twin anemia polycythemia syndrome not treated with fetoscopic laser treatment
- Multiple pregnancy with more than two fetuses
- Decision documented to give palliative neonatal care

If all eligibility criteria are met, the parents are asked for consent. In case of consent and imminent birth before 30 weeks of gestation, randomization will follow.

Note that once an infant is randomized, it remains in the study even when it later becomes apparent that one or more eligibility criteria were not met. The only exception is that when the 30th week of gestation is reached after randomization, the allocated intervention will not be carried out and the infant will be excluded. In the unlikely event that labor or practical preparations for a caesarean section start just before 30 weeks, and the team and parents are prepared to carry out the randomized strategy and birth takes place at 30 + 0 weeks, the exclusion is not deemed appropriate.

Randomization, blinding, and treatment allocation

Infants are 1:1 randomized to either PBCC or TBCC. Allocation is stratified by gestational age (< 27 + 0 and ≥ 27 + 0 weeks) and treatment center using random permutated block (4–8) sizes. Concealment of allocation

is ensured by using the randomization process of Castor Electronic Data Capture (Amsterdam, The Netherlands, www.castoredc.com), an electronic data capture system. Blinding of the allocation arm during the intervention is not possible in this trial. Independent assessors who verify the primary outcome are blinded for treatment allocation.

In the case of twin vaginal birth, both infants are randomized to the same group. In the case of caesarean section for twins, it is technically not possible at this moment to perform PBCC in both infants. After consent, both infants are included; the first infant always receives standard treatment without randomization. The second infant is randomized to either PBCC or standard treatment.

Determination of sample size

The sample size is determined to detect an increase in intact survival due to treatment of 10% (from 72 to 82%) with 80% power and a test size (alpha) of 5%. The incidence of intact survival (72%) was calculated from recent historical databases of Leiden University Medical Centre and Erasmus Medical Centre. We have performed the sample size calculation as if a chi-square test would be used in the analysis to reduce the number of assumptions needed. The required sample size was 275 individuals in each arm. Because of the inclusion of twins and an anticipated 10% cross-over from the intervention group to the control group, we increased the sample size to 330 randomized participants in each arm, resulting in 660 randomizations in total. When there are 25% of twins of which 66% are delivered by a caesarean section, we anticipate the need for parental consent for circa 720 infants.

Statistical methods

The flow diagram in Fig. 1 shows the different study stages and expected patient flow. In the final report, a similar figure will be completed with the actual numbers, to clarify the number of infants not receiving the allocated intervention, lost to follow-up, or excluded from analysis for another reason. In case of treatment failure (defined as the number of participants in which abortion

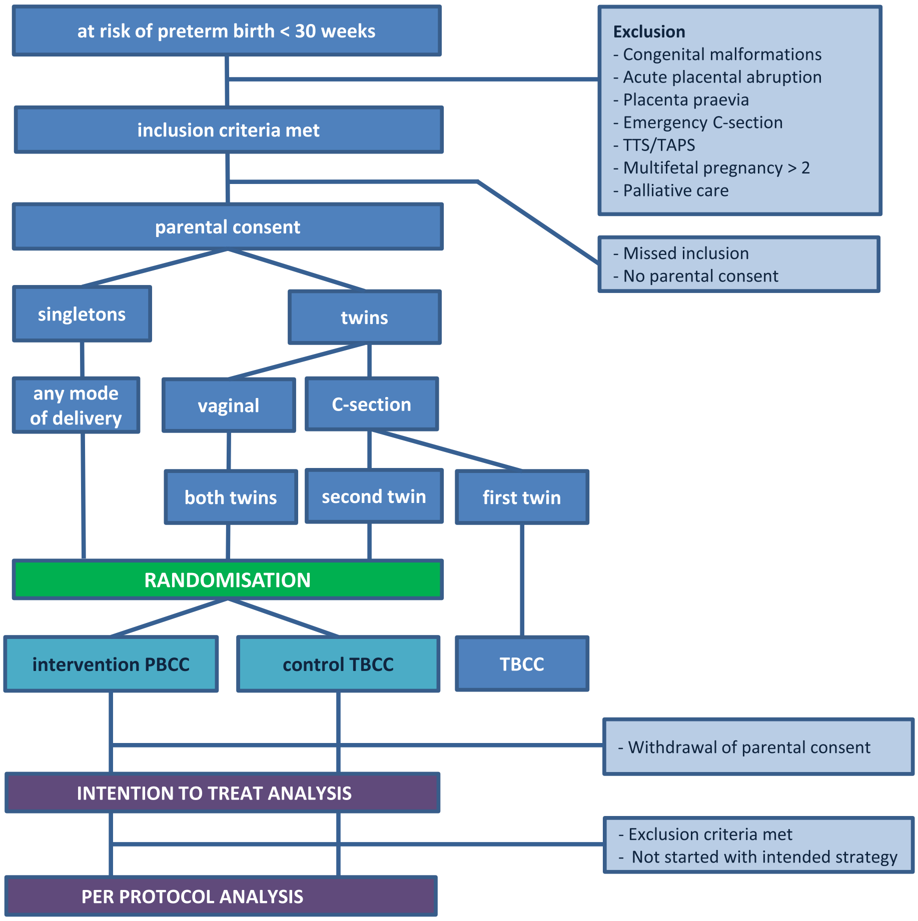

Fig. 1 Flow diagram of the ABC3 trial

of prescribed procedure (PBCC or TBCC) occurred), the reasons for abortion will be reported.

Handling of missing data

All possible efforts will be made to complete the datasets. Anticipating a (nearly) complete dataset, we plan to conduct all analyses on complete cases only. Infants for which one of the variables required in the analysis is missing will be excluded from that analysis. Completeness of cases is judged on an analysis-by-analysis basis and will be reported. We assume that missing data are likely missing not at random. As imputation may increase bias, we decided not to apply multiple imputation.

Infants for which consent is withdrawn are excluded from all analyses. We will report how often this occurred. If consent is withdrawn before discharge, while study recruitment is still running in that center, replacement will take place by inclusion and randomization of another infant.

Data handling

Data handling and monitoring have been described in detail in the study protocol [1].

Potential outliers are investigated. Extreme outliers, defined as being more than three times the interquartile range below the first quartile or more than three times the interquartile range above the third quartile, will be listed individually in a supplement to the main analyses. They will be excluded only if it can be reasonably assumed that they are due to an error in the data.

After completion, cleaning, and validation of the data, the dataset will be locked before the statistical analyses will be carried out. The lock for the final database will be applied once and will not be reversed, except in exceptional circumstances and only with agreement from the trial team.

Analysis sets

Intention-to-treat set

The primary analysis for this study is done on an intention-to-treat basis. This allows estimation of the realized (causal) benefit of implementing PBCC over TBCC. Furthermore, in this way, selection bias is minimized and the benefits of randomization are optimally used. The analysis set for these analyses consists of all infants that have been randomized, irrespective of actual treatment received, protocol violations, or exclusion criteria.

Per-protocol set

A secondary per-protocol analysis is performed to estimate the benefit of using PBCC (instead of merely having the intention to do so) over TBCC in infants that actually receive PBCC, in the target group. This set takes the intention-to-treat set as a basis, but excludes:

- infants who do not meet the inclusion criteria or do meet any of the exclusion criteria.
- infants who do not start with the intended strategy, for any reason. For example, in the case of randomization for PBCC in twins based on an expected vaginal birth, followed by a switch to a caesarean section, the first twin who will not receive PBCC will be excluded from the PP set.

Note that infants who switch to TBCC after initiating PBCC will be analyzed in the PBCC group to which they were randomized. We do not anticipate that infants randomized to the TBCC group receive the PBCC treatment.

An as-treated analysis, where the infants who do not start with intended PBCC are analyzed in the TBCC group, may introduce bias as cross-over is likely not random. Therefore, an as-treated analysis (as mentioned in the study protocol) is not deemed appropriate.

If birth took place at 30 + 0 weeks, the infant will remain in the dataset.

First twins' caesarean section set

The first twins from an anticipated caesarean section will be included as a separate group in the descriptive analyses, but are not included in any comparative analysis.

Descriptive analyses

Baseline variables of interest are gestational age at birth, birth weight, sex, maternal age and parity, maternal smoking, single or twin gestation, monochorionic or dichorionic placentation, small for gestational age (birthweight < P10), mode of birth, complications of pregnancy (preterm prelabor rupture of membranes, hypertensive disorders, chorioamnionitis), use of prenatal corticosteroids, and other maternal medication.

For both trial arms and the additional control group consisting of the first twins, we list the median (1st quartile; 3rd quartile) of continuous baseline variables and the observed frequencies (and percentages) of categorical baseline variables. No statistical test will be performed to compare these baseline variables.

Primary analyses

To compare the difference in primary outcome between the two arms, a logistic regression model will be estimated using generalized estimating equations (GEE) with an exchangeable working correlation matrix and non-robust standard errors, to account for the potential correlation in the outcome between siblings and infants within the same center. The response of this model is

intact survival at NICU discharge and the covariates are the treatment arm and gestational age.

We will report the number of infants that reached the primary endpoint for the PBCC or standard treatment arm as well as for the group of first twins. This will also be presented as a percentage together with a confidence interval.

We will also calculate the marginal absolute risk difference (ARD) for the intervention. This is done by calculating the predicted probability of the primary outcome under both arms for each individual in the intention-to-treat set. The ARD can now be estimated by taking the average of the difference between the arms. A confidence interval will be calculated using the bootstrap.

We will repeat the analyses for the per-protocol set (Table 1).

Secondary outcomes

Secondary outcomes to be explored are listed in Table 2. To explore competing risks with mortality, composite outcomes with mortality will be defined for each of the following four major secondary outcomes: IVH, BPD, NEC, and ROP. Additionally, treatment failure defined as abortion of prescribed procedure (PBCC or TBCC) and reasons for abortion are collected.

Short-term parental reported outcomes are based on questionnaires on parental perception and appreciation of the approach during birth and perinatal stabilization. These include the rating of 10 items (appreciation of the procedure, anxiety, satisfaction, maternal and newborn safety, contact with their newborn (general, visual, and tactile), size of the team present, provision of information), on a 5-point scale, as well as an open question.

Long-term outcomes will be assessed at the standard follow-up visits at 2 years of corrected age. These secondary outcomes will include mental and psychomotor neurodevelopmental outcomes and quality of life items [1]. Definitions and detailed descriptions of the analysis plan for these long-term outcomes are not clearly defined yet and therefore not part of this statistical analysis plan.

The cost-effectiveness of the intervention will be evaluated, based on quality-adjusted life years (QALYs) in relation to healthcare and non-health care costs during the follow-up period of 2 years. A more detailed description of the methods and planned analyses is described in the study protocol [1].

Secondary outcomes will be analyzed using generalized estimating equations (GEE) as is done for the primary outcome. For binary variables, a Bernoulli probability function and logit-link function are used; for continuous outcomes, a Gaussian probability function and an identity-link are used, while for count outcomes a Poisson distribution with a log link is used. We will allow for overdispersion and will use negative binomial or normal models, in case the assumed mean–variance relation that was assumed looks to be strongly violated as determined by visual inspection. For the purpose of the analyses, the parental reported outcomes measured on a 5-point scale are considered to be continuous. For sparse binary outcomes (quasi-)separation might occur which may result in biased or even nonfinite parameter estimates. In these cases, we will apply Firth bias correction. Furthermore, the working correlation is simplified by not taking twins into account (GEE is robust to misspecification of the working correlation). The analyses of the secondary outcomes aim to generate new hypotheses. As no multiplicity adjustments will be applied, results on secondary outcomes will be reported as explorative.

Subgroup analyses

To explore differences in response to the intervention in different categories of infants, subgroup analysis will be done, using the intention-to-treat set. Separate analyses will be carried out for two groups based on gestational age, mode of birth, and sex of the infant (Table 3). The logistic model used will be expanded by an interaction term between the grouping variable and the treatment. We will compare treatment arms within both subgroups and also report the *p*-value for the test of a different treatment effect between the subgroups. Note that regardless of the outcome of this test, the interaction term will remain in the model.

An exploratory analysis is planned on the learning curve of this new intervention. We hypothesize that the practice of the intervention may improve with more experience within a center. Therefore, we will study the interaction between the number of passed interventions and the intervention effect on the primary outcome. This will be added to the earlier described model.

Table 1 Mock table of the reporting on the results of the analysis of the primary outcome of the ABC3 study

Analyses set	Intact survival PBCC	Intact survival TBCC	*p*-value	Odds ratio	Absolute risk difference
Intention to treat	*N* (%)	*N* (%)	*p*-value	OR (95% CI)	ARD (95% CI)
Per protocol	*N* (%)	*N* (%)	*p*-value	OR (95% CI)	ARD (95% CI)

PBCC Physiological-based cord clamping, *TBCC* Time-based cord clamping, *N* Number, *OR* Odds ratio, *CI* Confidence interval, *ARD* Absolute risk difference

Table 2 List of secondary outcomes, including the levels of measurement

	Unit of measurement	*Categorial*	*Additional*
Maternal and placental variables			
Estimated total blood loss	mL		
Postpartum hemorrhage > 1000 mL		Yes/No	
Rupture of umbilical cord		Yes/No	
Placental weight	Gram		
Surgical site infection after caesarean section		Yes/No	
Variables related to stabilization at birth			
Support during transition		Yes/No	
Use of supplemental oxygen		Yes/No	
Use of continuous positive airway pressure		Yes/No	
Use of positive pressure ventilation		Yes/No	
Use of endotracheal ventilation		Yes/No	
Use of chest compressions		Yes/No	
Use of epinephrin		Yes/No	
Number of lung inflations			Total number
Number of sustained inflation			Total number
Maximum FiO2 administered			Fraction
Apgar scores at 1, 5, and 10 min		1–10	
Time to stabilization	Minutes and seconds		
Cord clamping time	Minutes and seconds		
Arterial umbilical cord pH			
Infant variables			
Temperature at admission	Degrees Celsius		
Hemoglobin (< 24 h)	mmol/L		
Hematocrit	L/L		
Polycythemia (venous hematocrit > 0.65 l/l)		Yes/No	
Intubation (< 72 h)		Yes/No	
Respiratory distress syndrome		Yes/No	
Surfactant therapy		Yes/No	
Pneumothorax		Yes/No	
Pulmonary hemorrhage		Yes/No	
Pulmonary interstitial emphysema		Yes/No	
Oxygen requirement (> 21%)	Days		
Bronchopulmonary dysplasia [6, 7]		Yes/No	Stage
Volume expansion (< 72 h)		Yes/No	
Inotropic use (< 72 h)		Yes/No	
Persistent ductus arteriosus requiring treatment		Yes/No	Treatment
Highest bilirubin level	µmol/l		
Hyperbilirubinemia requiring therapy		Yes/No	Treatment
Culture-proven early-onset sepsis		Yes/No	
Culture-proven late-onset sepsis		Yes/No	Total number
Meningitis		Yes/No	
Necrotizing enterocolitis [8]		Yes/No	Stage, treatment
Focal intestinal perforation		Yes/No	
Red blood cell transfusion		Yes/No	Total number
Intraventricular hemorrhage [9]		Yes/No	Stage
Post-hemorrhagic ventricular dilatation		Yes/No	Treatment
Periventricular venous infarction [9]		Yes/No	
Periventricular leukomalacia [10]		Yes/No	Stage

Table 2 (continued)

	Unit of measurement	*Categorial*	*Additional*
Cerebellar hemorrhage		Yes/No	
Seizures		Yes/No	
Retinopathy of prematurity [11]		Yes/No	Stage, treatment
Retinopathy of prematurity plus disease [11]		Yes/No	
Mortality at 28 days postnatal age		Yes/No	
Mortality at 36 weeks PMA		Yes/No	
Mortality at hospital discharge		Yes/No	
Length of NICU stay	Days		
Length of hospital stay	Days		
Weight, length, and head circumference at discharge	Gram, cm		

FiO2 Fraction of inspired oxygen, *PMA* Post-menstrual age, *NICU* Neonatal intensive care unit

Table 3 Mock table showing reporting on the results of the subgroup analyses

Subgroup	Intact survival PBCC	Intact survival TBCC	*p*-value	Odds ratio	Absolute risk difference
**Gestational age**					
< 27 + 0 weeks	*N* (%)	*N* (%)	*p*-value	OR (95% CI)	ARD (95% CI)
≥ 27 + 0 weeks	*N* (%)	*N* (%)	*p*-value	OR (95% CI)	ARD (95% CI)
Interaction effect gestational age			*p*-value	OR (95% CI)	
**Mode of birth**					
Vaginal	*N* (%)	*N* (%)	*p*-value	OR (95% CI)	ARD (95% CI)
CS	*N* (%)	*N* (%)	*p*-value	OR (95% CI)	ARD (95% CI)
Interaction effect mode of birth			*p*-value	OR (95% CI)	
**Sex**					
Boys	*N* (%)	*N* (%)	*p*-value	OR (95% CI)	ARD (95% CI)
Girls	*N* (%)	*N* (%)	*p*-value	OR (95% CI)	ARD (95% CI)
Interaction effect sex			*p*-value	OR (95% CI)	

PBCC Physiological-based cord clamping, *TBCC* Time-based cord clamping, *CS* Caesarean section, *N* Number, *OR* Odds ratio, *CI* Confidence interval, *ARD* Absolute risk difference

More exploratory post hoc (subgroup) analyses may be conducted; these will be clearly marked as such. Based on the exploratory nature of the subgroup analyses, no multiplicity adjustment is made to *p*-values and confidence intervals.

Interim analyses

An external data monitoring committee (DMC) monitors safety outcomes and provides recommendations regarding the continuation or premature termination of the trial. The independent DMC consists of an obstetrician, neonatologist, statistician, and epidemiologist, all experienced in clinical research and not involved in the trial. No interim analyses concerning efficacy are performed. We planned two interim statistical analyses on safety during the course of this study, after approximately 25% and 50% of the total required infants completed their primary outcome. The only stopping condition is based on safety. The decision to stop or continue is made by the trial team based on the advice of the DMC.

In the interim analyses, a reduced set of baseline variables (gestational age, birthweight, and sex) and outcomes is studied. To study safety, the primary outcome of the study was analyzed using GEE as described above. Secondary outcomes studied are infant death, NEC Bell's stage ≥ 2, severe cerebral injury, maternal blood loss, maternal blood loss > 1000 mL, infant temperature (°C), hypothermia (< 32 °C), and rupture of the umbilical cord. Unlike the final analysis, stratification factors are not taken into account, as strata are expected to be small in the interim stages. The causes of death for deceased infants are summarized in line listing for inspection by the DMC. Because the trial will never be prematurely stopped based on efficacy, no alpha-spending is required.

Significance levels and multiplicity adjustments

A significance level of 5% will be used for all tests. No formal multiplicity adjustment will be used; however, allowance for multiple testing will be made in the discussion of the results of the descriptive analyses, secondary outcomes, and subgroups.

Software

We will use R version 3.5.0 (or later) for the interim and final analysis [12].

Trial reporting

When reporting the results of the trial, we will follow the principles laid out in the CONSORT statement [13].

Trial status

The study is conducted according to the principles of the Declaration of Helsinki, good clinical practice guidelines, and the Dutch law (Medical Research Involving Human Subjects Act).

The trial is funded by The Netherlands Organisation for Health Research and Development (project number 852001902). AtP is a recipient of an NWO innovational research incentives scheme (VIDI 91716428). RK received a grant from the Sophia Children's Hospital Foundation (Rotterdam, S17-14). This project was sponsored by the Gisela Thier Fund (Leiden).

The study protocol was reviewed and approved by the Medical Ethical Committee of the LUMC, on Dec 19, 2018. The trial was registered at clinicaltrials.gov NCT03808051 on January 17, 2019, followed by the first infant being recruited at the Leiden University Medical Centre. Since then, nine other centers have started recruiting. All centers have been monitored as planned. Also as planned, two interim analyses have been run, upon which the DMC gave consent to continue the study. Inclusion was completed in October 2022. Completion of the short-term outcome dataset is expected in the second quartile of 2023. Data analyses on the short-term outcomes will not be started before the short-term dataset has been locked, and this statistical analysis plan has been submitted for publication in a peer-reviewed journal.

Abbreviations

ABC	Aeration, Breathing, Clamping
DCC	Delayed cord clamping
IVH	Intraventricular hemorrhage
NEC	Necrotizing enterocolitis
PVL	Periventricular leukomalacia
BPD	Bronchopulmonary dysplasia
TBCC	Time-based cord clamping
PBCC	Physiological-based cord clamping
NICU	Neonatal intensive care unit
PMA	Postmenstrual age
CS	Caesarean section
QALY	Quality-adjusted life years
GEE	Generalized estimating equations
OR	Odds ratio
CI	Confidence interval
N	Number
ARD	Absolute risk difference
(S)AE	(Serious) adverse event
DMC	Data monitoring committee
MREC	Medical Research Ethics Committee

Supplementary Information

The online version contains supplementary material available at https://doi.org/10.1186/s13063-024-08014-y.

Supplementary Material 1.

Acknowledgements

Not applicable.

Protocol version

This manuscript of the Statistical Analysis Plan (unrevised Version 1.0, 23-04-2023) is part of Version 2.2 of the Study Protocol, dated 28 April 2021.

Authors' contributions

SPW, RK, and MJV wrote the statistical analysis plan and all authors participated in reviewing the SAP. All authors are involved in gathering, reviewing, and cleaning the data in the trial. SPW wrote the first draft of the manuscript. All authors participated in reviewing and editing the manuscript. All authors have read and approved the final manuscript.

Funding

The trial is funded by The Netherlands Organisation for Health Research and Development (project number 852001902). AtP is a recipient of an NWO innovational research incentives scheme (VIDI 91716428). RK received a grant from the Sophia Children's Hospital Foundation (Rotterdam, S17-14). This project was sponsored by the Gisela Thier Fund (Leiden).

Availability of data and materials

Study data from the trial described in this manuscript will be retained and archived for a minimum of 15 years after study completion as per national regulations. There are no plans for publicly sharing the trial data. All data generated and/or analyzed during the trial are available from the corresponding author upon reasonable request.

Declarations

Ethics approval and consent to participate

The study is and will be conducted according to the principles of the Declaration of Helsinki and in accordance with the Dutch law (Medical Research Involving Human Subjects Act). The study protocol (study ID number NL67770.058.18) is evaluated and approved by the MREC of the LUMC (reference P18.218). Parental consent is required before trial participation.

Consent for publication

Not applicable.

Competing interests

The authors declare that they have no competing interests. The equipment used in this trial was either designed and built by LUMC (for 3 participating centers) or purchased from Concord Neonatal B.V. (Leiden, The Netherlands; for 6 participating centers). ABtP, SBH, and AHvK are members of the Scientific Advisory Board of Concord Neonatal B.V., but have no financial relationship or support. The company has no role in the design of the study; in the collection, analysis, or interpretation of data; or in the writing of the manuscript.

Author details
[1]Department of Intensive Care Neonatology and Children, Division of Neonatology, Sophia Children's Hospital, Erasmus MC University Medical Center, P.O. Box 2060, Rotterdam 3000 CB, The Netherlands. [2]Department of Biostatistics, Erasmus MC University Medical Centre Rotterdam, Rotterdam, The Netherlands. [3]Department of Pediatrics, Division of Neonatology, Leiden University Medical Center, Leiden, The Netherlands. [4]Department of Obstetrics, Leiden University Medical Center, Leiden, The Netherlands. [5]Athena Institute, VU University, Amsterdam, The Netherlands. [6]Department of Obstetrics and Gynaecology, Erasmus MC University Medical Center, Rotterdam, The Netherlands. [7]The Ritchie Centre, Hudson Institute of Medical Research, Monash University, Clayton, VIC, Australia. [8]Department of Neonatology, Emma Children's Hospital, Amsterdam UMC, University of Amsterdam and Vrije Universiteit Amsterdam, Amsterdam, The Netherlands. [9]Amsterdam Reproduction & Development, Amsterdam, the Netherlands. [10]Department of Pediatrics, Division of Neonatology, Radboud University Medical Center, Radboud Institute for Health Sciences, Amalia Children's Hospital, Nijmegen, The Netherlands. [11]Department of Neonatology, Isala Women and Children's Hospital, Zwolle, The Netherlands. [12]Department of Pediatrics, Division of Neonatology, Beatrix Children's Hospital, University Medical Center Groningen, Groningen, The Netherlands. [13]Department of Pediatrics, Maxima Medical Center, Veldhoven, The Netherlands. [14]Department of Pediatrics, Maastricht University Medical Center, Maastricht, The Netherlands. [15]Department of Neonatology, Wilhelmina Children's Hospital, University Medical Center Utrecht, Utrecht, The Netherlands.

Received: 16 May 2023 Accepted: 22 February 2024
Published online: 04 March 2024

References

1. Knol R, Brouwer E, van den Akker T, DeKoninck PLJ, Lopriore E, Onland W, et al. Physiological-based cord clamping in very preterm infants: the Aeration, Breathing, Clamping 3 (ABC3) trial-study protocol for a multicentre randomised controlled trial. Trials. 2022;23(1):838.
2. Madar J, Roehr CC, Ainsworth S, Ersdal H, Morley C, Rudiger M, et al. European resuscitation council guidelines 2021: newborn resuscitation and support of transition of infants at birth. Resuscitation. 2021;161:291–326.
3. Sweet DG, Carnielli VP, Greisen G, Hallman M, Klebermass-Schrehof K, Ozek E, et al. European consensus guidelines on the management of respiratory distress syndrome: 2022 update. Neonatology. 2023;120(1):3–23.
4. Bhatt S, Alison BJ, Wallace EM, Crossley KJ, Gill AW, Kluckow M, et al. Delaying cord clamping until ventilation onset improves cardiovascular function at birth in preterm lambs. J Physiol. 2013;591(8):2113–26.
5. Knol R, Brouwer E, Vernooij ASN, Klumper F, DeKoninck P, Hooper SB, et al. Clinical aspects of incorporating cord clamping into stabilisation of preterm infants. Arch Dis Child Fetal Neonatal Ed. 2018;103(5):F493–7.
6. Jobe AH, Bancalari E. Bronchopulmonary dysplasia. Am J Respir Crit Care Med. 2001;163(7):1723–9.
7. Walsh MC, Wilson-Costello D, Zadell A, Newman N, Fanaroff A. Safety, reliability, and validity of a physiologic definition of bronchopulmonary dysplasia. J Perinatol. 2003;23(6):451–6.
8. Walsh MC, Kliegman RM. Necrotizing enterocolitis: treatment based on staging criteria. Pediatr Clin North Am. 1986;33(1):179–201.
9. Volpe JJ. Intraventricular hemorrhage in the premature infant–current concepts. Part II Ann Neurol. 1989;25(2):109–16.
10. de Vries LS, Eken P, Dubowitz LM. The spectrum of leukomalacia using cranial ultrasound. Behav Brain Res. 1992;49(1):1–6.
11. International Committee for the Classification of Retinopathy of P. The International Classification of Retinopathy of Prematurity revisited. Arch Ophthalmol. 2005;123(7):991–9.
12. R Core Team. A language and environment for statistical computing. R Foundation for Statistical Computing. [https://www.R-project.org/].
13. Moher D, Hopewell S, Schulz KF, Montori V, Gotzsche PC, Devereaux PJ, et al. CONSORT 2010 explanation and elaboration: updated guidelines for reporting parallel group randomised trials. BMJ. 2010;340:c869.

Publisher's Note

Springer Nature remains neutral with regard to jurisdictional claims in published maps and institutional affiliations.
