# Supplementary material for: Physiological versus time based cord clamping in very preterm infants (ABC3): a parallel-group, multicentre, randomised, controlled superiority trial
Source: Lancet Reg Health Eur. 2024 Dec 4;48:101146. doi: 10.1016/j.lanepe.2024.101146 (PMC11664066; doi:10.1016/j.lanepe.2024.101146)
Supplement: C1 Protocol_ABC3_v2.2 [file mmc4.pdf]

Physiological-based cord clamping in very preterm infants: a multicentre randomised controlled trial

Aeration, Breathing, Clamping (ABC) project, study 3

RESEARCH PROTOCOL

PROTOCOL TITLE

Physiological-based cord clamping in preterm infants: a multicentre randomised controlled trial

**Protocol ID**	**Physiological-based cord clamping in preterm infants**
**Short title**	**The Aeration Breathing and Clamping (ABC) project: study 3**
**Version**	**2.2**
**Date**	**28-04-2021**
**Coordinating investigator/project leader**	**Prof. Dr. A.B. te Pas**<br>Department of Paediatrics, Division of Neonatology<br>Leiden University Medical Centre<br>J6-S, PO Box 9600<br>2300 RC Leiden<br>The Netherlands<br>a.b.te_pas@lumc.nl
**Principal investigator(s)**	**Prof. Dr. A.B. te Pas**<br>Department of Paediatrics, Division of Neonatology<br>Leiden University Medical Centre<br>J6-S, PO Box 9600<br>2300 RC Leiden<br>The Netherlands<br>a.b.te_pas@lumc.nl<br><br>**Drs. R. Knol**<br>Department of Paediatrics, Division of Neonatology<br>Erasmus MC – Sophia's Children Hospital<br>PO Box 2060<br>3000 CB Rotterdam<br>The Netherlands<br>r.knol@erasmusmc.nl
**Co-Investigators**<br>**LUMC, Leiden**	 **Prof. Dr. E. Lopriore**<br>Department of Paediatrics, Division of Neonatology<br>Leiden University Medical Centre<br>e.lopriore@lumc.nl<br><br>**Dr. T. van den Akker**<br>Department of Obstetrics<br>Leiden University Medical Centre<br>t.h.van_den_akker@lumc.nl<br><br>**Dr. M.E. van den Akker-van Marle**<br>Department of Biomedical Data Sciences<br>Section Medical Decision Making<br>Leiden University Medical Centre

	m.e.van_den_akker-van_marle@lumc.nl
	**Dr. L. van Bodegom-Vos**<br>Department of Biomedical Data Sciences<br>Section Medical Decision Making<br>Leiden University Medical Centre<br>l.van_bodegom-vos@lumc.nl
**Erasmus MC, Rotterdam**	**Dr. P. DeKoninck**<br>Department of Obstetrics<br>Erasmus Medical Centre, Rotterdam<br>p.dekoninck@erasmusmc.nl
	**Dr. M.J. Vermeulen**<br>Department of Paediatrics, Division of Neonatology<br>Erasmus Medical Centre, Rotterdam<br>m.j.vermeulen@erasmusmc.nl
	**Prof. Dr. I.K.M. Reiss**<br>Department of Paediatrics, Division of Neonatology<br>Erasmus Medical Centre, Rotterdam<br>i.reiss@erasmusmc.nl
**Monash University, Melbourne, Australia**	**Prof. Dr. S.B. Hooper**<br>The Ritchie Centre<br>Hudson Institute of Medical Research<br>Clayton, Victoria, Australia<br>Stuart.hooper@monash.edu
	**Dr. G.R. Polglase**<br>The Ritchie Centre<br>Hudson Institute of Medical Research<br>Clayton, Victoria, Australia<br>Graeme.polglase@monash.edu
**Netherlands Neonatal Research Network (NNRN)**	**Dr. W. Onland**<br>Director of NNRN, neonatologist<br>Amsterdam University Medical Centre<br>w.onland@amc.uva.nl
**AMC, Amsterdam**	**Prof. Dr. A.H.L.C. van Kaam**<br>Department of Neonatology<br>Amsterdam University Medical Centre<br>a.h.vankaam@amc.uva.nl
	**Dr. M. Oudijk**<br>Department of Obstetrics<br>Amsterdam University Medical Centre<br>m.oudijk@amc.uva.nl
**UMC, Utrecht**	**Dr. J. Dudink**<br>Department of neonatology<br>University Medical Centre Utrecht<br>j.dudink@umcutrecht.nl

**Isala Klinieken, Zwolle**	**Drs. E.E.M. Mulder**<br>Department of Neonatology<br>Isala Medical Centre Zwolle<br>e.e.m.mulder@isala.nl
**UMCG, Groningen**	**Dr. C.V. Hulzebos**<br>Department of Neonatology<br>University Medical Centre Groningen<br>c.v.hulzebos@umcg.nl
**Radboud UMC, Nijmegen**	**Dr. W.P. de Boode**<br>Department of Neonatology<br>Radboud University Medical Centre Nijmegen<br>willem.deboode@radboudumc.nl
**MMC, Veldhoven**	**Dr. P. Andriessen**<br>Department of Neonatology<br>Maxima Medical Centre Veldhoven<br>p.andriessen@mmc.nl<br><br>**Dr. S.J. van Sambeeck**<br>Department of Neonatology<br>Maxima Medical Centre Veldhoven<br>SamJanneke.van.Sambeeck@mmc.nl
**MUMC, Maastricht**	**Drs. M.E. van der Putten**<br>Department of Neonatology<br>Maastricht University Medical Centre<br>mayke.vander.putten@mumc.nl
**VUMC, Amsterdam**	**Prof. Dr. A.H.L.C. van Kaam**<br>Department of Neonatology<br>Amsterdam University Medical Centre<br>a.h.vankaam@vumc.nl<br><br>**Dr. S. Prins**<br>Department of Neonatology<br>Amsterdam University Medical Centre<br>s.prins@vumc.nl
**Universitätsklinikum Carl Gustav Carus Dresden, Germany**	**Dr.med. L. Mense**<br>Department of Neonatology & Pediatric Intensive Care<br>Universitätsklinikum Carl Gustav Carus an der Technischen Universitat Dresden<br>Lars.mense@uniklinikum-dresden.de<br><br>**Prof. Dr. M. Ruediger**<br>Department of Neonatology & Pediatric Intensive Care<br>Universitätsklinikum Carl Gustav Carus an der Technischen Universitat Dresden<br>Mario.ruediger@uniklinikum-dresden.de

**Sponsor (in Dutch: verrichter/opdrachtgever)**	**Leiden University Medical Centre**<br>Albinusdreef 2<br>2300 RC Leiden<br>The Netherlands
**Subsidising party**	**ZonMW**
**Independent expert(s)**	**Dr. A.A.W. Roest**<br>Department of Paediatrics<br>Leiden University Medical Centre<br>J6-S, PO Box 9600<br>2300 RC Leiden<br>The Netherlands<br>a.roest@lumc.nl
**Laboratory sites**	***None***
**Pharmacy**	***None***

PROTOCOL SIGNATURE SHEET

Name	Signature	Date
**Head of Department:**<br>***Prof.dr. E.H.H.M. Rings***<br>***Head of department of Paediatrics***		
**Head of Division:**<br>***Prof.dr. E. Lopriore***<br>***Head of Division of Neonatology***		
**Coordinating Investigator/Project leader:**<br>***Prof.dr. A.B. te Pas***<br>***Paediatrician-Neonatologist***		

TABLE OF CONTENTS

1. INTRODUCTION AND RATIONALE 13
2. OBJECTIVES 16
3. STUDY DESIGN 16
4. STUDY POPULATION 16
4.1 Population (base) 16
4.2 Inclusion criteria 16
4.3 Exclusion criteria 16
4.4 Sample size calculation 17
5. TREATMENT OF SUBJECTS 19
5.1 Investigational treatment 19
5.2 Use of co-intervention 19
6. METHODS 20
6.1 Study parameters/endpoints 20
6.1.1 Main study parameter 20
6.1.2 Other study parameters 20
6.2 Randomisation, blinding and treatment allocation 22
6.3 Study procedures 22
6.4 Withdrawal of individual subjects 24
6.5 Replacement of individual subjects after withdrawal 24
6.6 Follow-up of subjects withdrawn from treatment 25
6.7 Premature termination of the study 25
7. SAFETY REPORTING 26
7.1 Temporary halt for reasons of subject safety 26
7.2 Adverse Events (AEs) and Serious Adverse Events (SAEs) 26
7.3 (Context-specific) SAE reporting 26
7.4 Follow-up of adverse events 28
7.5 Annual safety report 28
7.6 Data Safety Monitoring Board (DSMB) 28
8. STATISTICAL ANALYSIS 29
8.1 Analysis 29
8.2 Interim analyses 29
9. ETHICAL CONSIDERATIONS 30
9.1 Regulation statement 30
9.2 Recruitment and consent 30
9.3 Benefits and risks assessment, group relatedness 30
9.4 Compensation for injury 30
10. ADMINISTRATIVE ASPECTS, MONITORING AND PUBLICATION 32
10.1 Handling and storage of data and documents 32
10.2 Monitoring and Quality Assurance 32
10.3 Amendments 32
10.4 Annual progress report 32

10.5 Temporary halt and (prematurely) end of study report 33
10.6 Public disclosure and publication policy 33
11. STRUCTURED RISK ANALYSIS 34
12. ORGANISATION 34
12.1 Trial Steering Committee (TSC) 34
12.2 Trial Management Group (TMG) 34
12.3 Data Safety Monitoring Board (DSMB) 34
12.4 Study Monitoring 35
13. REFERENCES 36

LIST OF ABBREVIATIONS AND RELEVANT DEFINITIONS

**ABR**	**ABR form, General Assessment and Registration form, is the application form that is required for submission to the accredited Ethics Committee (In Dutch, ABR = Algemene Beoordeling en Registratie)**
**CA**	**Competent Authority**
**CCMO**	**Central Committee on Research Involving Human Subjects; in Dutch: Centrale Commissie Mensgebonden Onderzoek**
**CV**	**Curriculum Vitae**
**DSMB**	**Data Safety Monitoring Board**
**GCP**	**Good Clinical Practice**
**IC**	**Informed Consent**
**METC**	**Medical research ethics committee (MREC); in Dutch: medisch ethische toetsing commissie (METC)**
**(S)AE**	**(Serious) Adverse Event**
**Sponsor**	**The sponsor is the party that commissions the organisation or performance of the research, for example a pharmaceutical company, academic hospital, scientific organisation or investigator. A party that provides funding for a study but does not commission it is not regarded as the sponsor, but referred to as a subsidising party.**
**TMG**	**Trial Management Group**
**TSC**	**Trial Steering Committee**
**Wbp**	**Personal Data Protection Act (in Dutch: Wet Bescherming Persoonsgevens)**
**WMO**	**Medical Research Involving Human Subjects Act (in Dutch: Wet Medisch-wetenschappelijk Onderzoek met Mensen**
**ABC**	**Aeration of the lung, establish Breathing, and then Clamping of the cord**
**ICC**	**Immediate Cord Clamping**
**DCC**	**Delayed Cord Clamping**
**TBCC**	**Time-Based Cord Clamping**
**PBCC**	**Physiological-Based Cord Clamping**
**NICU**	**Neonatal Intensive Care Unit**

**CPAP**	**Continuous Positive Airway Pressure**
**PPV**	**Positive Pressure Ventilation**
**GA**	**Gestational Age**
**PMA**	**Post Menstrual Age**
**PNA**	**Post Natal Age**
**IVH**	**Intraventricular Haemorrhage**
**NEC**	**Necrotizing Enterocolitis**
**PPH**	**Post-Partum Haemorrhage**

SUMMARY

Rationale: Preterm infants could benefit from placental transfusion (blood transfer from the placenta to the infant) when cord clamping is delayed. A recent meta-analysis comparing delayed cord clamping (DCC) with immediate cord clamping in preterm infants showed a decrease in mortality and a trend towards fewer intraventricular haemorrhages. However, in most studies DCC was performed at a fixed time point of 30-60 seconds after birth, while placental transfusion is only complete after 3 minutes. In addition, preterm infants needing immediate interventions for stabilisation or resuscitation were clamped immediately and excluded from analysis, while these infants might benefit the most of DCC.

While the rationale of all cord clamping studies was to allow for placental transfusion, studies in preterm lambs recently demonstrated that delaying cord clamping beyond ventilation onset also prevents a significant drop in cardiac output. This approach avoided large disturbances in systemic and cerebral haemodynamics and concomitant bradycardia and hypoxia. Avoiding these adverse effects may decrease the risk of cerebral injury and hypoxia-related diseases such as necrotizing enterocolitis and associated rates of mortality and morbidity in preterm infants.

These adverse effects described above may be avoided when preterm infants are first stabilised with an intact cord and clamping is postponed until the infant is considered cardiopulmonary stable. We called this approach ‘physiological-based cord clamping’ (PBCC), since the moment of cord clamping is based on the clinical condition of the infant. Until now PBCC was practically not possible since the cord needed to be clamped in order to move the infant to the resuscitation table. The recent development of a new purpose-built resuscitation table (the Concord) in Leiden makes it possible to provide all the necessary interventions for cardiopulmonary stabilisation, while the cord remains intact. A recent safety and feasibility study in preterm infants showed that PBCC using the Concord was feasible and safe. We observed a reduction of the incidence of bradycardia and hypoxia at birth, supporting the earlier observed increased stability during haemodynamic transition in animal studies. In addition, average cord clamping time exceeded 4 minutes, which allows preterm infants to benefit from a more complete and optimal placental transfusion.

We hypothesize that PBCC in preterm infants at birth will lead to an increase in intact survival (survival without cerebral injury and/or necrotizing enterocolitis) when compared to standard care.

Objective: To compare the effect of umbilical cord clamping after cardiopulmonary stabilisation (Physiological Based Cord Clamping; PBCC) to standard care (Time Based Cord Clamping; TBCC) on intact survival and health care costs in preterm infants.

Study design: Multicentre randomised controlled trial, parallel design, superiority trial.

Study population: Infants < 30 weeks of gestation

Intervention: PBCC; stabilisation of the infant with the umbilical cord intact and only clamp the cord when the infant is cardiopulmonary stable using a purpose-built resuscitation table, the Concord.
Control: TBCC; infants are clamped first and then moved to the resuscitation table for further interventions for cardiopulmonary stabilisation. Clamping is time-based and performed immediately or delayed at 30-60 sec, depending on the clinical condition of the infant, as recommended in current international guidelines.

Main study parameters/endpoints: Primary outcome will be intact survival at NICU discharge, defined as survival without cerebral injury (intraventricular haemorrhage ≥ grade 2

and/or periventricular leukomalacia ≥ grade 2 and/or periventricular venous infarction) and/or necrotizing enterocolitis (Bell stage ≥ 2).

Nature and extent of the burden and risks associated with participation, benefit and group relatedness: Most very preterm infants breathe insufficiently at birth and require assisted stabilisation. Very preterm infants have a high risk of mortality, cerebral injury and necrotizing enterocolitis and could benefit from PBCC using the Concord, which is designed to perform all standard care for stabilisation while the infant remains attached to the cord. Based on animal studies and our safety and feasibility studies we expect no additional burden or risk over and above the risk related to prematurity.

1. INTRODUCTION AND RATIONALE

Every year 14.000 preterm infants are born in the Netherlands, of whom 2500 are very preterm infants (< 32 weeks of gestation).(1) Very preterm infants have a high mortality rate and survivors have an increased risk for long term neurodevelopmental sequelae and health related problems.(2) At least 50% of all neonatal deaths are due to preterm birth and half of the children with severe disabilities are born preterm.(3)

The care for preterm infants has improved considerably and mortality has decreased. Unfortunately, neonatal morbidity has not changed significantly and therefore new interventions to improve neonatal outcomes are urgently needed. Most research and innovation have focused on optimizing treatment after the infant is admitted to the neonatal intensive care unit (NICU). However, there is convincing evidence that interventions applied in the delivery room in the first 10 minutes after birth, may have long-lasting impact on neonatal outcome.(4-9) This introduces opportunities to further improve care for and outcome of preterm infants.

Most preterm infants fail to aerate their immature lungs and need respiratory support at birth for survival. The practical problem is that in the current setting of the delivery rooms, the umbilical cord needs to be clamped first before interventions for cardiopulmonary stabilisation can be started. This approach compromises cardiovascular function and placental transfusion.(10, 11) The resulting hypoperfusion and hypoxia will subsequently lead to the use of more aggressive interventions to stabilise the condition of the infant, increasing the risk of mortality, cerebral injury and necrotizing enterocolitis (NEC).(6, 12-14) This will not only lead to a longer NICU admission and more readmissions, but also increase healthcare costs related to disabilities later in life.

There is strong evidence suggesting that preterm infants benefit from placental transfusion (blood transfer from the placenta to the infant) when cord clamping is delayed.(15) A recent meta-analysis comparing delayed cord clamping (DCC) with immediate cord clamping (ICC) in preterm infants showed increased haematocrit, less blood transfusions, a decrease in mortality and a trend towards less intraventricular haemorrhages (IVH).(15) In addition, a Cochrane Systematic Review showed a decreased incidence of NEC after DCC.(16) However, in most studies DCC was performed using a fixed time of 30-60 seconds, while placental transfusion is only complete after 3 minutes.(17) Waiting longer is considered unfeasible, as no cardiopulmonary support can be given during the period of delaying. In addition, preterm infants needing immediate interventions for stabilisation or resuscitation were clamped immediately and not included, while these infants have the highest risks of complications.

While the rationale of all cord clamping studies was placental transfusion, recent studies in preterm lambs demonstrated that delaying cord clamping until after ventilation onset prevents a significant drop in cardiac output.(18) Large disturbances in systemic and cerebral haemodynamics and concomitant bradycardia and hypoxia after ICC were avoided.(11) This finding may explain the bradycardia and hypoxia that is common in preterm infants after cord clamping. Avoiding these adverse effects may decrease the risk of cerebral injury and hypoxia-related diseases such as NEC and associated rates of mortality and morbidity.(4, 6)

The adverse effects could be avoided when preterm infants are first stabilised with the cord intact and not clamped until the infant is cardiopulmonary stable. We have called this approach ‘physiological-based cord clamping’ (PBCC) as the moment of cord clamping is based on the clinical condition of the infant.(19) In contrast, the current cord clamping approach is based on a fixed time-point (time-based cord clamping (TBCC)). Until now PBCC was practically not possible as the cord needed to be clamped first to move the infant to the resuscitation table. We have now solved this issue by designing a new purpose-built resuscitation table (the Concord) in Leiden. This table makes it possible to provide all the necessary interventions for cardiopulmonary stabilisation, while the cord remains intact. In a recent safety and feasibility study in preterm infants, PBCC using the Concord was feasible and safe.(*Brouwer et al,*

submitted) We observed less bradycardia and hypoxia at birth, supporting the more stable haemodynamic transition observed in the animal studies. In addition, the average cord clamping time was over 4 minutes, which allows preterm infants to benefit from optimized placental transfusion.

Mechanisms/Pathophysiology

Very preterm infants have an increased risk of mortality and long-term sequelae due to the complications of prematurity. The incidence of NEC is 5-10%, with case fatality rates between 12% and 30% and those who survive have a high risk for long-term problems.(20) Preterm infants also have a high risk of IVH (> 20%) and cerebral white matter damage, leading to cerebral palsy, motor and coordination problems, epilepsy and severe cognitive impairment.(21, 22) Cardiopulmonary instability is known as an important risk factor for the incidence of cerebral damage and NEC.

Bradycardia and hypoxia at birth are associated with a 3-fold increased risk of developing IVH and death.(6) In addition, when infants are bradycardic or hypoxic more vigorous resuscitation is needed, which is associated with a 4-fold increased risk for IVH.(5) Hypotension and assisted ventilation both are associated with increased risk of NEC.(23) Both anemia and hypotension have been associated with an increased risk of NEC and IVH.(23-25) In addition, hypotension in the first days after birth increases the risk of periventricular leukomalacia (PVL) and death.(26, 27) We hypothesize that PBCC will result in optimal placental transfusion and cardiopulmonary stability at birth, leading to improved clinical outcomes.(16)

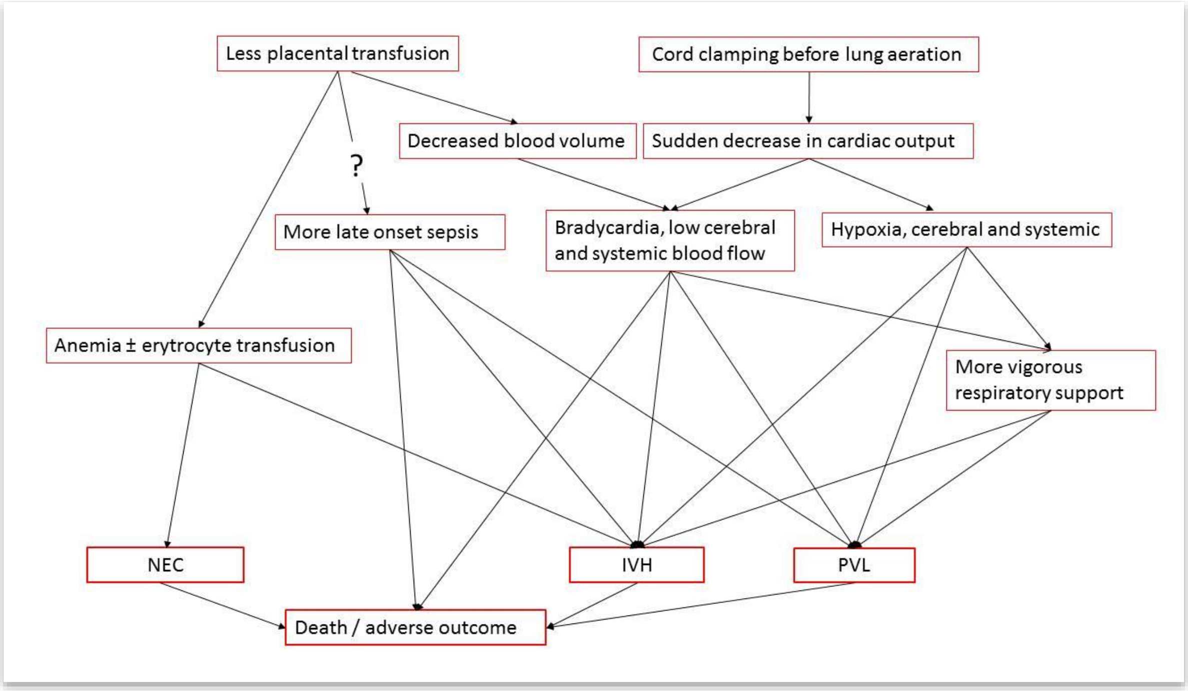

Figure 1. Pathophysiology of immediate cord clamping.

Preterm infants surviving without IVH or NEC will have a shorter stay in the NICU, will be readmitted less frequently and will have fewer long-term neurodevelopmental sequelae and health related problems when compared to infants who survive with one or more preterm related morbidities.(28, 29) Reduction of NEC and IVH will have considerable positive impact on healthcare costs.(30-32) There are no national studies performed relating the morbidities

to (re)admission days, but based on local databases, very preterm infants (< 30 weeks of gestation) who have cerebral injury and/or NEC stay an average of at least 8 days longer in the NICU and have 7 more readmission days in the first year in comparison to infants with intact survival.

Hypothesis

In this study we will test the hypothesis that PBCC will lead to an increase in intact survival (survival without significant cerebral injury and/or necrotizing enterocolitis) when compared to standard approach (TBCC).

2. OBJECTIVES

Primary Objective: To compare the effect of umbilical cord clamping after cardiopulmonary stabilisation (PBCC) to time-based cord clamping (TBCC) on intact survival in preterm infants.

Secondary Objective: To compare the effect of umbilical cord clamping after cardiopulmonary stabilisation (PBCC) to time-based cord clamping (TBCC) on health care costs in preterm infants.

3. STUDY DESIGN

Multicentre randomised controlled trial, parallel design, superiority trial.

4. STUDY POPULATION

4.1 Population (base)

Very preterm infants, born at < 30 weeks of gestation in one of the participating centres.

4.2 Inclusion criteria

Inclusion criteria in this study are:

- Infants born at a gestational age below 30 weeks in a participating centre.
- Parental consent (see 9.2).

4.3 Exclusion criteria

A potential subject who meets any of the following criteria will be excluded from participation in this study:

- Significant congenital malformations.
- Signs of acute placental abruption.
- Total placenta praevia, anterior placenta praevia or invasive placentation (accreta/percreta).
- Birth by emergency caesarean section (ordered to be executed within 15 minutes).
- Twin gestation with signs of Twin Transfusion Syndrome or Twin Anaemia Polycythemia Syndrome not treated with fetoscopic laser treatment.
- Multiple pregnancy > 2 (triplets or higher order).
- Decision documented to give palliative neonatal care.

In case of twin delivery by caesarean section it is not possible to perform PBCC in both infants. Both infants will be included: the first infant will receive standard treatment and the second infant will be randomised to either PBCC or standard treatment.

4.4 Sample size calculation

Estimation of the background incidence of the primary outcome in preterm infants below 30 weeks of gestation is based on historical databases in recent years of Leiden University Medical Centre and Sophia Children's Hospital. Intact survival at discharge from the hospital (defined as survival without significant cerebral injury (IVH ≥ grade 2 and/or PVL ≥ grade 2 and/or periventricular venous infarction) and/or NEC ≥ Bell stage 2) is estimated at 72%.

Estimation of the effect size of the intervention cannot be based on earlier trails, as this will be the first human clinical trial on efficacy. PBCC has been performed in preterm lamb studies and in a pilot study on feasibility and safety, but the number of patients in the pilot study was small and a control group was lacking.

We estimated the effect of the intervention based on the following arguments:

- In the animal and the pilot study, PBCC has led to less bradycardia, less cerebral hypoperfusion, fewer fluctuations in haemodynamic function and less hypoxia at birth, which may all be related to the primary outcome according to observational studies.
- We will study a high-risk population of preterm infants with a gestational age below 30 weeks, who usually need stabilisation/resuscitation after birth. Infants needing stabilisation/resuscitation, who have the highest risk for complications, may benefit most from PBCC and placental transfusion.
- Using the PBCC approach allows for more complete placental transfusion, which may enhance the beneficial effects as seen earlier in the DCC versus ICC trials. It was shown in a recent meta-analysis, including all studies comparing DCC to ICC, that mortality was significantly reduced (RR 0.68, [0.52, 0.90]), the need for blood transfusions was reduced (RR 0.81 [0.74, 0.87]) and there was a trend towards lower incidence of IVH (RR 0.87 [0.75, 1.00]).(15) We expect a larger effect of PBCC as compared to DCC because in most DCC studies cord clamping was performed at 30 to 60 seconds and infants requiring resuscitation or stabilisation were excluded.

Taking this all into account we consider an absolute increase of 10% of the intact survival (from 72% to 82%) to be a realistic effect that is clinically relevant.

The sample size calculation was based on the following:

- We calculated that at least 550 (275 in each arm) infants are needed to detect an absolute difference of 10% (0.72 to 0.82 intact survival), with 80% power, at a significance level of 0.05.
- According to the protocol, vaginally born twins will be randomised in pairs. Based on the shared environment and (partly) shared genetic background, high correlation in outcome can be expected between both twins. Both will be included in the analysis, but for the sample size calculation we considered one twin pair equally informative as 1 singleton participant. Thus, compensation for 30 pairs is included in the calculation.
- In case of caesarean section, for practical reasons first twins are automatically assigned to control treatment and will not be randomised. Their data will be recorded according to the study protocol, but their number is not included in the estimated sample size (N=60).
- Based on the feasibility study, we expect 10% of patients to be clamped earlier than PBCC protocol prescribes and do not expect that to happen in the control group. The primary analysis will be based on an intention-to-treat principle. To preserve sufficient power we increased the number needed per arm with another 10%.
- Based on experience in earlier trials and on historical local databases from the NICU's in Leiden and Rotterdam we assumed the following:
  - 20% of eligible patients will meet the exclusion criteria.
  - 40% of those who meet the inclusion criteria are not included for logistical reasons or parental refusal.
  - 75% of fetuses are singletons, 25% part of twins.

- 33% of the twins will be born vaginally, 66% via caesarean section.

Based on the above we aim to include 330 neonates in each arm. Figure 2 illustrates the expected numbers needed to achieve that.

Assuming that in each of the 10 participating centres approximately 100 infants < 30 weeks of gestation are born each year and delay in start of recruitment in centres, we expect to reach full study recruitment within 2 years.

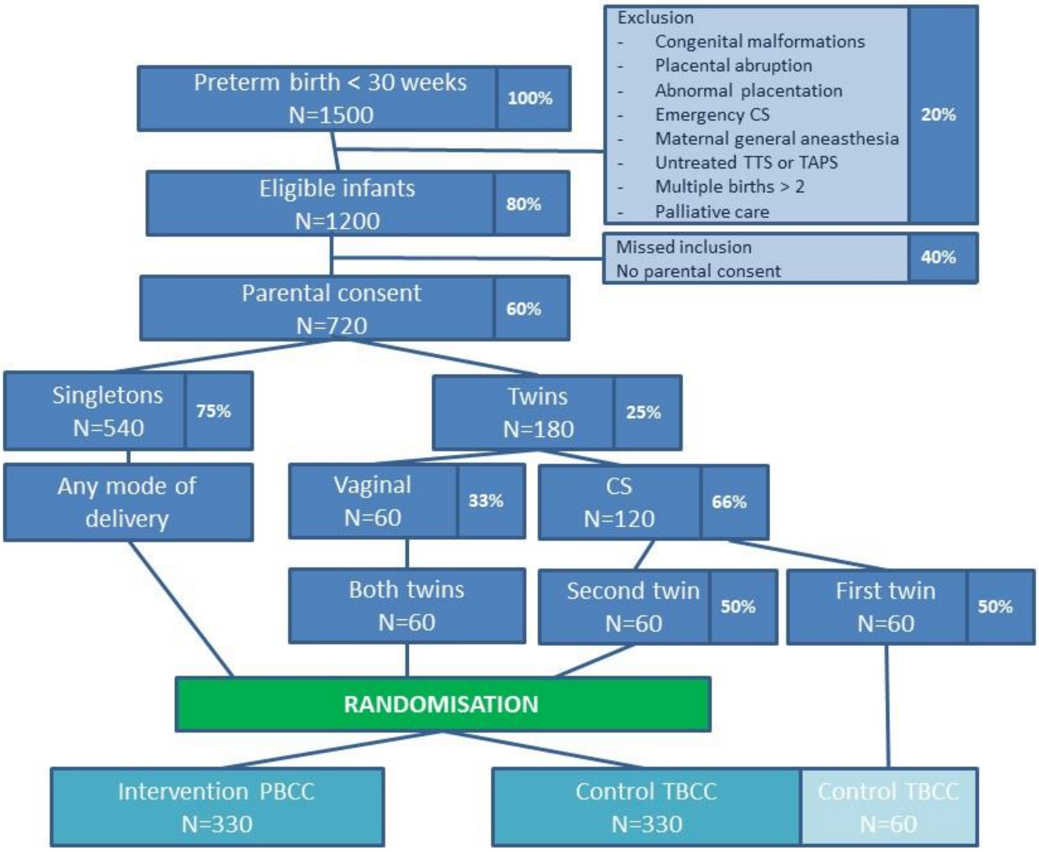

Figure 2. Flow chart illustrating the randomisation plan showing estimated numbers needed to include 660 participants in each arm.

5. TREATMENT OF SUBJECTS

5.1 Investigational treatment

Preterm infants randomised to the intervention group will be stabilised according to PBCC. As soon as the infant is born, the infant will be placed on the Concord and respiratory support will be started according to the local resuscitation guidelines. The umbilical cord will not be clamped until the infant is stabilised. Stable is defined as the establishment of heart rate greater than 100 bpm and oxygen saturation above 85% while using supplemental oxygen lower than 40%. The maximum time of cord clamping is 10 minutes and prior to cord clamping a trial of weaning from PPV to CPAP is performed. With the exception that the infant is stabilised close to the mother and the cord is clamped in a later stage, the infant will receive standard treatment.

5.2 Use of co-intervention

All randomised patients will receive normal interventions as part of the stabilisation (e.g. heat loss prevention, respiratory support), which is standard care. The interventions are given according to international resuscitation guidelines.(33) Small differences in standard care can be present between units when local protocols deviate from the international guidelines. All performed delivery room interventions will be recorded in the CRF.

6. METHODS

6.1 Study parameters/endpoints

6.1.1 Main study parameter

Primary outcome:

The dichotomous outcome intact survival at NICU discharge is defined as survival without major cerebral injury (IVH ≥ grade 2 and/or PVL ≥ grade 2 and/or periventricular venous infarction) and/or NEC ≥ Bell stage 2.

Cerebral injury will be assessed by ultrasonography. Cerebral ultrasounds will be performed according to the national protocol as part of standard care (cerebral ultrasound at postnatal day 1, 3, 7, 14 and 28, and then every 2 weeks until NICU discharge). They will be performed by the attending ultrasound specialist, who is trained and experienced. All cerebral ultrasound recordings will be reviewed and scored by an independent researcher blinded for the treatment allocation. For the grading of IVH and PVL we will use the definitions of Volpe and De Vries, respectively.(34, 35)

NEC will be diagnosed according to internationally used modified Bell's staging criteria, requiring radiographical signs of pneumatosis intestinalis and/or portovenous gas to be classified as stage 2 or higher.(36, 37) The diagnosis of NEC will be ascertained by having it reviewed by an independent researcher blinded for treatment allocation. Cases of spontaneous focal intestinal perforation, defined as isolated perforation in a normal-appearing bowel without features of NEC such as pneumatosis intestinalis or necrosis, are not classified as NEC.(38) Cases without pneumatosis but with (sub)total intestinal necrosis confirmed by surgeon (during laparotomy) or pathologist (tissue biopsy or post-mortem) are defined as NEC stage 3.(39)

6.1.2 Other study parameters

Baseline characteristics:

Demographic details and patient characteristics that will be collected from the medical files include maternal age, parity, maternal smoking, indicators of socio-economic status (SES), maternal pre-pregnancy BMI, gestational age (based on known first day of last menstruation if menstrual cycle was regular (28 days +/- 5days) or based on early fetal ultrasonography), birth weight, sex, single or twin gestations, monochorionic or dichorionic, small for gestational age, mode of delivery, complications of pregnancy (premature rupture of membranes, hypertensive disorders, chorioamnionitis, gestational diabetes), use of antenatal corticosteroids (single dose, full dose), other maternal medication.

Secondary outcomes:

1. *Procedure related*
   - Details of the stabilisation at birth (cord clamping time) and interventions (respiratory support (PPV and/or CPAP), max pressures, max FiO2, intubation)
   - Treatment failure defined as abortion of prescribed procedure (intervention or control) and reasons for abortion.
   - Infant temperature at admission to the NICU

- Highest infant haemoglobin levels within 24 hours of age
- Occurrence of polycythemia (venous haematocrit > 0.65)

2. *Neonatal outcomes, short-term*
    - Apgar scores
    - Echocardiography measurement: ductal flow ratio at 1 hour of age (only LUMC)
    - Blood pressure, at 1 hour of age (only LUMC)
    - Intubation in first 72 hours
    - Infant Respiratory Distress Syndrome (IRDS)
    - Use of surfactant
    - Intravascular volume expansion in first 72 hours
    - Inotropes in first 72 hours
    - Pneumothorax
    - Persistent ductus arteriosus for which medical intervention or surgical ligation is necessary
    - Duration of phototherapy
    - Proven early onset sepsis (clinical suspicion with positive blood culture).
    - Proven late onset sepsis (clinical suspicion with positive blood culture > 72 hours after delivery).
    - Necrotizing enterocolitis ≥ grade 2
    - Surgical NEC: Necrotizing enterocolitis requiring surgical intervention (also if the patient was too unstable to undergo surgery).
    - Spontaneous focal intestinal perforation
    - Number of red blood cell transfusions
    - Intraventricular haemorrhage ≥ grade 2
    - Periventricular leukomalacia ≥ grade 2
    - Periventricular venous infarction
    - Bronchopulmonary dysplasia, including severity(40, 41)
    - Retinopathy of prematurity, including severity and treatment
    - Mortality at 28 days postnatal age (PNA), 36 weeks postmenstrual age (PMA) and at hospital discharge
    - Length of stay in NICU
    - Length of stay in hospital

3. *Maternal outcomes*
    - Maternal blood loss
    - Postpartum haemorrhage > 1000 ml
    - Placental weight
    - Surgical site infection after caesarean section

4. *Neonatal outcomes, long-term*
    - Long-term neurodevelopmental outcomes assessed at 2 years corrected age:
        - Bayley Scales of Infant Development III (BSID-III-NL)
        - Mental Developmental Index (MDI)
        - Psychomotor Developmental Index (PDI)
        - Cerebral palsy and severity of CP
        - Hearing loss requiring hearing aids
        - Blindness
        - Behavioural problems (CBCL)

All clinical primary and secondary endpoints are measured as standard usual care and will be derived from the medical charts of the patients by the investigators. No additional investigations will take place, except for the measurements of the ductal flow ratio together with blood pressure at 1 hour of age, for infants that are included at the LUMC. The ductal flow ratio measurements will be made by our researcher if feasible.

The parents and the caregivers will be asked to fill in a questionnaire concerning their perception and appreciation of the approach during birth and the stabilisation stage.

5. *Secondary outcomes, cost-effectiveness:*
   - Total (re)-admission days
   - Other healthcare use (e.g. outpatient visits, home therapy (such as tube feeding), paramedical care, GP visits)
   - Non-medical costs children (e.g. day-care)
   - Productivity losses parents
   - Quality of life children, using the TAPQOL and PedsQL (generic score) questionnaires (adapted for young children)
   - Quality of life parents, using EQ-5D-5L questionnaire

Healthcare costs include all health care use during follow-up e.g. NICU-days, readmissions, treatments, outpatient visits, and GP visits. Non-health care costs consist of lost productivity costs of parents from paid and unpaid work and costs of (specialized) day-care for children. Health care use and absence from work will be assessed with diaries filled out by parents every 6 months. For the valuation of health care, reference prices published in the Dutch costing guidelines will be used.(42) Costs of absenteeism from paid work will be calculated using the friction cost method.

The TAPQOL and EQ-5D-5L will be completed at 6 months, and after that every 6 months during follow-up until 2 years corrected age. The PedsQL (generic score) questionnaire will be completed at 18 months and 2 years corrected age. Utilities will be calculated from the questionnaires using so-called tariffs.(43-45) Using the area-under-the-curve method for the utility scores obtained for infants and parents, the QALY outcome for infants and parents will be obtained for the trial-based cost-effectiveness analysis. Furthermore, utility values will be used as input in the model for the lifetime cost-effectiveness analysis.

6.2 Randomisation, blinding and treatment allocation

Infants will be 1:1 randomised to either PBCC or standard treatment. Allocation will be stratified by gestational age (24-26+6 and 27-29+6 weeks) and by treatment centre using random permutated block (4-8) sizes. Concealment of allocation will be ensured by using the randomisation process of Castor EDC, an electronic data capture system. During office hours the researchers will randomise using a personalized account. During non-office hours a general account is available for the neonatal fellow or neonatal consultant in charge of the procedure who will randomise using Castor EDC. Blinding of the allocation arm in this study is not possible.

In case of twin vaginal delivery, both infants will be randomised to the same group. In case of twin caesarean section, it is technically not possible at this moment to perform PBCC in both infants. After consent, both infants will be included; the first infant will always receive standard treatment and the second infant will be randomised to either PBCC or standard treatment.

6.3 Study procedures

Prior to the start of the study, all caregivers involved in delivery room care will be trained in using the Concord (by Concord Neonatal BV, Leiden, the Netherlands) for PBCC, for which instruction videos and workshops have been developed. A standard operating procedure (SOP) has been developed enabling close collaboration between the obstetrical and neonatal team. The Concord is a mobile table that can be used to perform PBCC in preterm infants born

after vaginal birth or caesarean section. All neonatal caregivers involved are trained experts and accredited for neonatal resuscitation. Centres will adhere to their local resuscitation guidelines for stabilisation, which are based on international guidelines.

Parents will be approached for participation antenatally to ensure time for explanation, questions and decision-making. The procedure for consent is described in paragraph 9.2.

The infant will be randomised to either **intervention (PBCC) or standard approach (TBCC).**

PBCC

The Concord will be placed next to the bed of the mother on the left side and all equipment will be checked before the 2nd stage of labour has started or prior to caesarean section. In both groups, stabilisation will be performed as soon as the infant is placed on the table and according to the local resuscitation guidelines.

When an infant is randomised to PBCC, also the standard resuscitation table will be set and prepared for use. In any case the attending neonatologist and/or obstetrician considers that PBCC should not be performed or interrupted, the infant can be taken to the standard resuscitation table for (further) stabilisation.

Timing of cord clamping, performing PBCC:

Stabilisation of the infant is performed while the cord is intact and the cord will be clamped after the infant is cardiopulmonary stable. Stable is defined as the establishment of heart rate greater than 100 bpm and oxygen saturation above 85% while using supplemental oxygen lower than 40%. The maximum cord clamping time is 10 minutes and prior to cord clamping a trial of weaning from PPV to CPAP is performed. With the exception that the infant is stabilised close to the mother and the cord is clamped in a later stage, the infant will receive standard treatment.

Summary of SOP PBCC procedure:

- The Concord is placed on the left side of the bed of the mother.
- After the infant is born, the obstetrician holds the baby.
- The platform of the table is placed as close as possible to the mother's pelvis to make sure that stretching of the umbilical cord will not occur.
- The infant is placed on the platform and receives stabilisation and heat loss prevention according to standard guidelines. The nurse places the oximeter probe on the right wrist of the baby.
- In case of vaginal birth, the mother is able to touch and stimulate the infant during stabilisation. The nurse communicates with the parents to explain the ongoing procedures.
- The cord is clamped as soon as the infant is cardiopulmonary stable (defined as the establishment of a heart rate > 100 bpm and oxygen saturation above 85% while using supplemental oxygen < 40%).
- To ensure optimal placental transfusion the minimum time before clamping is 3 minutes.
- If the infant does not reach the criteria for being stable, the maximum cord clamping time is 10 minutes.
- After clamping, the platform will be withdrawn and placed next to the bed of the mother.
- Uterotonic drugs are administered immediately after cord clamping.
- The infant will be prepared and transferred to the transport incubator.

In case of twins and vaginal birth, the same definition of the moment of cord clamping will be used. Clamping in the first infant will be performed sooner if the second infant of the twins is about to be born. The first infant will then be transferred to the standard resuscitation table, so that the second twin can be stabilised on the Concord.

TBCC

Preterm infants are clamped first and then moved to the standard resuscitation table for further treatment and intervention needed for cardiopulmonary stabilisation. Clamping is time based and performed immediately or delayed at 30-60 seconds, depending on the clinical condition of the infant. The Dutch national consensus guideline, based on international resuscitation guidelines, will be used to determine timing of cord clamping to ensure homogeneity between centres. Uterotonic drugs are administered immediately after cord clamping.

<u>Equipment used</u>

The Concord

The Concord is specially designed to provide full standard care in stabilisation of preterm infants at birth while the cord remains intact. The working plate can be placed very close to the mother so every preterm infant, independent of the length of the umbilical cord, can receive PBCC. A slit is incorporated in the working plate in which the umbilical cord can be placed without stretching the cord. The table is provided with all equipment needed for stabilisation and resuscitation.

The Standard table

For standard care a standard resuscitation table is used, provided with all equipment that is needed for stabilisation and resuscitation. This table is often situated in the resuscitation room, which is a room next to the delivery room or operation theatre.

All centres will be offered the opportunity to have a respiratory function monitor built in the Concord, to record physiological parameters and video during stabilisation. This monitor (Advanced Life Diagnostics, Weener, Germany) depicts respiratory function, oxygen saturation, heart rate, FiO2 and a video using the NewLifeBox-R physiological recording system (Advanced Life Diagnostics, Weener, Germany).

6.4 Withdrawal of individual subjects

Parents and caregivers can leave the study at any time for any reason if they wish to do so without any consequences. The clinician can decide to stop the PBCC approach for urgent medical reasons and change to standard care. These infants are not withdrawn from the study and remain in follow up, as this is an outcome parameter. PBCC can be aborted immediately, when:

- an emergency occurs with mother or the second twin and more working space is needed for the obstetrical team.
- full cardiac resuscitation for the infant is needed.
- maternal blood loss is excessive according to the obstetrical team and administration of uterotonic drugs is needed immediately.

6.5 Replacement of individual subjects after withdrawal

When parents choose to withdraw their infant for any reason before NICU discharge, another infant will be included and randomised in the study. When parents choose to withdraw their

infant for any reason after NICU discharge, we will ask their consent for using the so-far registered data.

6.6 Follow-up of subjects withdrawn from treatment

All infants included in the study will be included in routine follow-up until a corrected age of 2 years. Neurodevelopmental follow-up after discharge is standard care for all preterm infants < 30 weeks of gestation born in the Netherlands. In case of withdrawal from the study before the end of the intervention, infants will be analysed according to the intention-to treat-principle. Also, a per protocol analysis will be performed.

6.7 Premature termination of the study

The study will be terminated when serious adverse events occur significantly more often in the intervention group than in the control group. An external, independent Data Safety Monitoring Board (DSMB) will monitor the study on safety aspects and if necessary recommend termination of the study (see also Chapter 7.6 of this protocol and the DSMB Charter), based on the planned interim analyses.

7. SAFETY REPORTING

7.1 Temporary halt for reasons of subject safety

In accordance to section 10, subsection 4, of the WMO, the sponsor will suspend the study if there is sufficient ground that continuation of the study will jeopardise subject health or safety. The sponsor will notify the accredited METC without undue delay of a temporary halt including the reason for such an action. The study will be suspended pending a further positive decision by the accredited METC. The investigator will take care that all subjects' parents or caregivers are kept informed.

7.2 Adverse Events (AEs) and Serious Adverse Events (SAEs)

Adverse events are defined as any undesirable experience occurring to a subject during a clinical trial, whether or not considered related to the intervention. The study population consists of critically ill preterm infants, with a high incidence of serious and non-serious complications, which are inherent to their vulnerable condition and unrelated to the intervention which is under evaluation in this trial. Context-specific SAEs are defined (see 7.3). In addition to the context-specific SAEs, and for analysis purposes, only the following complications will be recorded in the Case Report Form as an AE:

- Congenital malformations
- Non-prematurity related treatments (medical or surgical)

A serious adverse event is any untoward medical occurrence or effect that:

- results in death;
- is life threatening (at the time of the event);
- requires hospitalization or prolongation of existing inpatients' hospitalization;
- results in persistent or significant disability or incapacity;
- is a congenital anomaly or birth defect (not applicable in this trial);
- other important events that may jeopardize the safety of the subject or may require intervention to prevent one of the outcomes listed above.

All SAEs will be reported, as described below (7.3), by the principle investigator to the Data Safety Monitoring Board (DSMB) and to the accredited METC that approved the protocol, according to the requirements of that METC. All SAEs in the centres using a non-CE approved Concord will be reported to the Health Care Inspectorate of the Ministry of Health, Welfare and Sport (Nederlandse Inspectie voor de Gezondheidszorg).

Any unforeseen SAEs that was life threatening or resulted in death and was directly related to the PBCC approach will be reported to the sponsor without undue delay after obtaining knowledge of the event. The sponsor will report the SAEs to the accredited METC that approved the protocol, within 7 days of first knowledge for SAEs that result in death or are life threatening and directly related to the PBCC approach, followed by a period of maximum of 8 days to complete the initial preliminary report.

Any unforeseen SAEs directly related to the PBCC approach and not considered life threatening or resulting in death are recorded in the Case Report Form and included in the yearly overview of the context-specific SAEs that will be presented to the DSMB and METC that approved the protocol.

7.3 (Context-specific) SAE reporting

This study population (critically ill preterm infants) has a high risk of serious complications (so-called “context-specific SAE’s”), which are inherent to their vulnerable condition and unrelated to the intervention which is under evaluation in this trial. These complications are included in the primary and secondary outcomes of this study and are recorded during NICU admission in the Case Report Form. This documentation will include date of diagnosis, classification/gradation of the complication and type of action taken if appropriate (with some complications a wait and see approach is warranted). Since most complications are highly interrelated and of longitudinal character, it may be impossible to indicate an exact date for the resolution or stabilisation of each specific diagnosis. In these cases, we will use the date of discharge from the NICU for this purpose. As long as the child is admitted to the NICU, the complication will be classified as ongoing.

Considering the above, immediate and individual reporting of all these condition related complications will not enhance the safety of study. This is also in accordance with CCMO regulations. Yearly, an overview of the context-specific SAEs for each treatment arm and ordered by organ system will be presented to the DSMB and METC. This overview will consist of the following information: name of the complication, date of diagnosis, classification/gradation of the complication, type of action taken, date of discharge or ongoing.

Stabilisation of preterm infants with the resuscitation table as close as possible to the mother has been performed before and is considered as safe.(46-48) We perform a similar method, but improved the approach by taking into consideration that 1) stretching of the umbilical cord is prevented, 2) full standard care for stabilisation can be provided and 3) extended infant monitoring is included. We do not expect that PBCC poses extra risks for the infant compared to the risks related to the stabilisation of the preterm infant. In our feasibility study no additional risks were reported.(*Brouwer et al, 2019*)

Nevertheless, we will include three `safety parameters' as SAE in this study that will be reported to the sponsor without undue delay after obtaining knowledge of the event. The sponsor will report these SAEs to the accredited METC that approved the protocol, within 7 days of first knowledge for SAEs that result in death or are life threatening and directly related to the PBCC approach, followed by a period of maximum of 8 days to complete the initial preliminary report. SAEs listed below that do not result in death or are not life threatening, will be reported within a period of maximum 15 days after the sponsor has first knowledge of the SAE:

- Severe hypothermia at NICU admission (defined by WHO as temperature < 32° C)
- Maternal post-partum haemorrhage (PPH, defined by WHO as blood loss > 1000 ml)
- Rupture of the umbilical cord during birth

The first two safety parameters are important because PBCC will result in later cord clamping times than used so far. An increased risk of hypothermia and PPH following PBCC has been suggested, although DCC-studies do not support these concerns.(16, 49) As mentioned before, we did not find problems concerning these parameters in our feasibility study.

An increased risk in rupture of the umbilical cord has also been suggested. So far, this has not been observed in the studies where stabilisation with intact umbilical cord has been performed. The standard risk for ruptured cord has only been described as a complication of controlled cord traction performed after cord clamping as part of active management of the third stage.(50) Recently the results of the CORD trial were published and the cord snapped in 1/137 patients (0.7%) where stabilisation was performed before cord clamping.(46) Based on the available data and the use of our adapted device, we do not expect an increased risk for cord rupture. In the unlikely event that a rupture would occur, cord clamping is performed immediately and stabilisation of the infant is continued.

All SAEs that are derived from the medical charts of the patients which do not meet the previous outlined criteria of a SAE related to the PBCC approach and the context-specific

SAEs are recorded in the Case Report Form and included in the yearly overview of the context-specific SAEs that will be presented to the DSMB and METC that approved the protocol.

7.4 Follow-up of adverse events

All AEs will be followed until they have abated, or until a stable situation has been reached. Depending on the event, follow up may require additional tests or medical procedures as indicated. According to the standard of care, all infants will participate in the usual NICU follow-up program. This program is targeted at evaluating and coordinating diagnostic procedures and treatment of all prematurity related problems, in close cooperation with regional and local paediatricians. SAEs will be reported till end of study, as defined in the protocol.

7.5 Annual safety report

The PI will submit an annual safety report to the DSMB, accredited METC, as well as the investigators of all participating centres. This safety report consists of:

- a list of all serious adverse reactions, along with an aggregated summary table of all reported serious adverse reactions.
- a report concerning the safety of the subjects, consisting of a complete safety analysis and an evaluation of the balance between the efficacy and the harmfulness of the intervention under investigation.

7.6 Data Safety Monitoring Board (DSMB)

An external Data Safety Monitoring Board (DSMB) will monitor only safety outcomes and will provide the Trial Steering Committee with recommendations regarding continuing or stopping the trial (for all patients or subgroups of patients) when approximately 25% and 50% of the anticipated outcome data are available. Data summaries for the DSMB will be prepared by a statistician who is not a member of the investigating team. The safety data will include, but not be restricted to, serious adverse events and the safety outcomes listed as secondary outcomes. The DSMB will not be blinded to the treatment allocation. If the DSMB recommends modification or cessation of the study protocol, this will be discussed with the Steering Committee, who will make the decision.

The DSMB will be composed of at least 4 individuals: a neonatologist with extensive knowledge of neonatal resuscitation, a biostatistician who has experience with trials and experience in previous DSMBs, a paediatrician who is experienced in methodology of conducting large trials and a representative of the parental association (VOC, Vereniging van Ouders van Couveusekinderen). The Trial Steering Committee will propose a detailed mandate and review this with the DSMB, from the outset. Identification and circulation of external evidence (e.g., from other trials/systematic reviews) is not the responsibility of the DSMB members. It is the responsibility of the PI to provide any such information to the DSMB.

The advice(s) of the DSMB will only be sent to the sponsor of the study. Should the sponsor decide not to fully implement the advice of the DSMB, the sponsor will send the advice to the reviewing METC, including a note to substantiate why (part of) the advice of the DSMB will not be followed.

8. STATISTICAL ANALYSIS

8.1 Analysis

Normally distributed data will be presented as mean ± standard deviations, not-normally distributed data as medians and (interquartile) ranges. Categorical data will be analysed using the Chi-square test. Continuous data will be analysed using the Student's *t* test or Mann-Whitney test as appropriate. Intention-to-treat analysis will be employed. Per protocol analysis will be employed as secondary analysis. The effect of PBCC on the primary outcome will be assessed by multi-variable logistic regression analysis including possible confounders. The effect of PBCC on the secondary outcomes will be assessed by multi-variable logistic regression analysis. Statistical significance is set at $p < 0.05$.

The economic evaluation from a societal perspective will consist of a trial-based cost-effectiveness analysis (costs per additional infant with intact survival) and a model-based cost-utility analysis (lifelong costs per QALY).

In the trial-based economic evaluation the effects of PBCC will be compared to TBCC and related to the difference in costs during the follow-up period of two years. Differences in mean costs and effects between strategies will be compared with two-sided bootstrapping. In a net-benefit analysis, costs will be related to the outcomes and presented in a cost-effectiveness acceptability curve. No discounting will be applied due to the short time horizon of the trial based economic evaluation. The evaluation will be performed from a societal perspective. Multiple imputation will be used for handling missing data.

In the model-based cost-utility analysis a decision tree model will be used to extrapolate the trial results to lifetime costs and QALYs for PBCC in comparison with usual care (TBCC). In this lifetime cost-utility analysis costs will be discounted at a percentage of 4% and effects at a percentage of 1.5%, according to the Dutch guidelines for health economic research.(51) Sensitivity analysis will be carried out for the most important input parameters.

Implementation scenarios will be evaluated from the relevant perspectives (societal, healthcare provider, healthcare insurer), in accordance with the Dutch BIA guidelines.(52)

8.2 Interim analyses

No interim analyses concerning efficacy will be performed. We will conduct two interim statistical analyses on safety during the course of this study, after approximately 25% and 50% of the total required patients have completed their primary outcome. The only stopping condition will be safety.

The results of the interim analyses will be judged by the DSMB. This board will act completely independently of the clinical investigators, including the Principal Investigators. The ultimate decision to stop the study will rest with the steering committee.

9. ETHICAL CONSIDERATIONS

9.1 Regulation statement

The study will be conducted according to the principles of the Declaration of Helsinki and in accordance with the Medical Research Involving Human Subjects Act (WMO).

9.2 Recruitment and consent

Antenatal consent will be obtained before birth if the mother is not in established labour and if time permits. In this situation, parents of an eligible infant will be informed by the attending obstetrician and/or neonatologist and asked for their written informed consent after they have read the information letter.

To prevent selection bias and increase generalisability we will strive to also include the most unexpected born preterm infants in the trial. For this reason, we will also approach parents for oral consent in case mother arrives in the hospital in full labour and tocolytic treatment is not an option. Parents will be informed on study goals and procedures and asked for oral consent. Written informed consent will be obtained as soon as possible afterwards.

We will not approach parents for consent in case of an emergency situation and immediate delivery (<15 minutes) is necessary or when approaching parents for consent is considered inappropriate. These infants will not be included in the study and deferred consent will not be used.

The parents of the infant can withdraw their consent at any moment during the study period.

9.3 Benefits and risks assessment, group relatedness

In this study most preterm infants need stabilisation at birth and might benefit from delayed cord clamping. Delayed cord clamping has been incorporated in international guidelines, mostly using a fixed time and delaying stabilisation until the cord has been clamped.(33, 53) Currently, studies are performed where stabilisation is performed while the cord remains intact using commercially available resuscitation tables. So far, stabilisation with the cord intact has been considered a safe approach, vaginally as well as during caesarean section.(46-48)

The infant has potentially more benefit from delaying cord clamping when PBCC is used. We do not expect that there is an added risk as the Concord is fully equipped for stabilisation and resuscitation. The feasibility study did not show any additional risks for the mother or the infant. Secondary outcomes include ‘safety parameters’ for the mother and the infant. While the mother will benefit for having her baby close to her and able to touch her baby, there is a risk that it will cause anxiety as interventions take place close to the parents. We will minimize this by communicating to the parents antenatally what to expect and during the stabilisation the nurse will communicate what happens during the stabilisation.

Stabilisation at birth of preterm infants occurs exclusively in this patient group. Any intervention to reduce the risks in these patient group therefore needs to be studied in this specific population.

9.4 Compensation for injury

The sponsor has a liability insurance which is in accordance with article 7 of the WMO. The sponsor has insurance with adequate coverage, which is in accordance with the legal requirements in the Netherlands (Article 7 WMO). This insurance provides cover for damage to research subjects through injury or death caused by the study. The insurance applies to the damage that becomes apparent during the study or within 4 years after the end of the study.

10. ADMINISTRATIVE ASPECTS, MONITORING AND PUBLICATION

10.1 Handling and storage of data and documents

Data management will be implemented according to Good Clinical Practice (GCP)-guidelines. Patient data will be entered by way of an eCRF in a central GCP proof internet-based database to facilitate on-site data-entry. Security is guaranteed with login names, login codes and encrypted data transfer. An experienced data manager will maintain the database and check the information in the database for completeness, consistency and plausibility.

The data of all subjects will be coded and this coding will not be retraceable to the individual patient. The key to this coding is safeguarded by the investigator. A limited number of people have access to the source data. These are the principal investigators, investigating personnel. Personal data are only processed by the researchers or by those who fall directly under their authority. In addition, the study monitor, quality assurance auditor, employees from the METC and the Health Care Inspectorate of the Ministry of Health, welfare and Sport (Nederlandse Inspectie voor de Gezondheidszorg) have access to the source data. All are subject to the pledge of confidentiality. Data will be stored for 15 years strictly confidential.

10.2 Monitoring and Quality Assurance

The study will be monitored by an experienced monitor throughout its duration by means of personal visits to the investigator’s facilities and through other communications (e.g., telephone calls, written correspondence). Monitoring visits will be scheduled at mutually agreed times periodically throughout the study and at frequency deemed appropriate for the study. For details we refer to the monitoring plan of the study.

These visits will be conducted to evaluate the progress of the study, ensure the rights and wellbeing of the subjects are protected, check that the reported clinical study data are accurate, complete and verifiable from source documents, and the conduct of the study is in compliance with the approved protocol and amendments, GCP and applicable national regulatory requirements. A monitoring visit will include a review of the essential clinical study documents (regulatory documents, CRFs, source documents, subject informed consent forms, etc.) as well as discussion on the conduct of the study with the Investigator and staff. The Investigator and staff should be available during these visits to facilitate the review of the clinical study records and resolve/document any discrepancies found during the visit.

10.3 Amendments

Amendments are changes made to the trial after a favourable opinion by the accredited METC has been given. All amendments will be notified to the METC that gave a favourable opinion.

All substantial amendments will be notified to the METC and to the competent authority. Non-substantial amendments will not be notified to the accredited METC and the competent authority, but will be recorded and filed by the sponsor.

10.4 Annual progress report

The investigator will submit a summary of the progress of the trial to the accredited METC once a year. Information will be provided on the date of inclusion of the first subject, numbers of

subjects included and numbers of subjects that have completed the trial, serious adverse events/ serious adverse reactions, other problems, and amendments.

10.5 Temporary halt and (prematurely) end of study report

The investigator will notify the accredited METC of the end of the study within a period of 8 weeks. The end of the study is defined as the last included infant. In case the study is ended prematurely, the investigator will notify the accredited METC within 15 days, including the reasons for the premature termination. Within one year after the end of the study, the investigator will submit a final study report with the results of the study, including any publications/abstracts of the study, to the accredited METC.

10.6 Public disclosure and publication policy

Results of the study will end up in multiple articles, which will be submitted for publication to peer-reviewed international medical journals, which will be defined later. The results will be presented at international conferences. In addition, the results of the study will be used to advice local, national and international resuscitation guidelines, which will benefit future patients.

11. STRUCTURED RISK ANALYSIS

For PBCC a new specially designed resuscitation table (the Concord) will be used. There are other resuscitation tables available that can be placed very close to mother and cord clamping could be postponed. However, stabilisation according to local resuscitation guidelines could not be guaranteed using the available tables. Adequate stabilisation is essential to establish lung aeration and adequate breathing. In collaboration with Neonatology, Obstetrics and Technical Engineering a new table has been developed that can provide this. The table is fully equipped to perform everything that is needed for stabilisation of the preterm infant.

All equipment needed for stabilisation that is attached to the table are approved medical devices. Additional information of the Concord and the risk analysis are included.

12. ORGANISATION

12.1 Trial Steering Committee (TSC)

The Trial Steering Committee is the main policy and decision-making committee of the study and has final responsibility for the scientific conduct of the study. The TSC will provide overall supervision of the trial and ensure that it is being conducted in accordance with the principles of Good Clinical Practice and the relevant regulations. It will be composed of representatives of the sponsor and of investigators of the participating centres. The TSC will meet by telephone conference monthly and face-to-face as need dictates, but at a minimum bi-annually.

The specific tasks of the Steering Committee are:

- Approval of the study protocol.
- Approval of necessary changes in the protocol based on considerations of feasibility.
- Act upon recommendations of the Data Safety Monitoring Board.
- Review performance reports of the study sites.
- Resolve operational problems brought before it by the project manager.
- Approval of study reports and papers for publication.

12.2 Trial Management Group (TMG)

The role of the TMG is to monitor all aspects of the conduct and progress of the trial, ensure that the protocol is adhered to and take appropriate action to safeguard participants and the quality of the trial itself. The TMG will include all who are responsible for the day-to-day management of the trial.

12.3 Data Safety Monitoring Board (DSMB)

An independent Data Safety Monitoring Board (DSMB) will be created specifically for this trial. The DSMB will act in advisory capacity to the TSC. See Paragraph 7.6 of this protocol and the DSMB charter for a description of the membership, tasks and responsibilities of the DSMB.

12.4 Study Monitoring

The study will be monitored by an experienced monitor throughout its duration by means of personal visits to the investigator’s facilities and through other communications. For the procedures see also the descriptions in paragraph 10.2.

13. REFERENCES

1. Pal van der SM, Pal van der-de Bruin KM. Call to action for Newborn Health. Vroeggeboorte in Nederland.: TNO; 2012 September. Report No.: TNO/CH 2012 R10565.
2. Harrison MS, Goldenberg RL. Global burden of prematurity. Semin Fetal Neonatal Med. 2016;21(2):74-9.
3. Blencowe H, Cousens S, Chou D, Oestergaard M, Say L, Moller AB, et al. Born too soon: the global epidemiology of 15 million preterm births. Reprod Health. 2013;10 Suppl 1:S2.
4. DeMauro SB, Roberts RS, Davis P, Alvaro R, Bairam A, Schmidt B, et al. Impact of delivery room resuscitation on outcomes up to 18 months in very low birth weight infants. J Pediatr. 2011;159(4):546-50 e1.
5. Mian Q, Cheung PY, O'Reilly M, Barton SK, Polglase GR, Schmolzer GM. Impact of delivered tidal volume on the occurrence of intraventricular haemorrhage in preterm infants during positive pressure ventilation in the delivery room. Arch Dis Child Fetal Neonatal Ed. 2018.
6. Oei JL, Finer NN, Saugstad OD, Wright IM, Rabi Y, Tarnow-Mordi W, et al. Outcomes of oxygen saturation targeting during delivery room stabilisation of preterm infants. Arch Dis Child Fetal Neonatal Ed. 2018;103(5):F446-F54.
7. Polglase GR, Miller SL, Barton SK, Kluckow M, Gill AW, Hooper SB, et al. Respiratory support for premature neonates in the delivery room: effects on cardiovascular function and the development of brain injury. Pediatr Res. 2014;75(6):682-8.
8. Schmolzer GM, Te Pas AB, Davis PG, Morley CJ. Reducing lung injury during neonatal resuscitation of preterm infants. J Pediatr. 2008;153(6):741-5.
9. Tarnow-Mordi W, Morris J, Kirby A, Robledo K, Askie L, Brown R, et al. Delayed versus Immediate Cord Clamping in Preterm Infants. N Engl J Med. 2017;377(25):2445-55.
10. Bhatt S, Alison BJ, Wallace EM, Crossley KJ, Gill AW, Kluckow M, et al. Delaying cord clamping until ventilation onset improves cardiovascular function at birth in preterm lambs. J Physiol. 2013;591(8):2113-26.
11. Polglase GR, Dawson JA, Kluckow M, Gill AW, Davis PG, Te Pas AB, et al. Ventilation onset prior to umbilical cord clamping (physiological-based cord clamping) improves systemic and cerebral oxygenation in preterm lambs. PLoS One. 2015;10(2):e0117504.
12. Goos TG, Rook D, van der Eijk AC, Kroon AA, Pichler G, Urlesberger B, et al. Observing the resuscitation of very preterm infants: are we able to follow the oxygen saturation targets? Resuscitation. 2013;84(8):1108-13.
13. Phillipos E, Solevag AL, Aziz K, van Os S, Pichler G, O'Reilly M, et al. Oxygen Saturation and Heart Rate Ranges in Very Preterm Infants Requiring Respiratory Support at Birth. J Pediatr. 2017;182:41-6 e2.
14. White LN, Thio M, Owen LS, Kamlin CO, Sloss S, Hooper SB, et al. Achievement of saturation targets in preterm infants <32 weeks' gestational age in the delivery room. Arch Dis Child Fetal Neonatal Ed. 2017;102(5):F423-F7.
15. Fogarty M, Osborn DA, Askie L, Seidler AL, Hunter K, Lui K, et al. Delayed vs early umbilical cord clamping for preterm infants: a systematic review and meta-analysis. Am J Obstet Gynecol. 2018;218(1):1-18.
16. Rabe H, Diaz-Rossello JL, Duley L, Dowswell T. Effect of timing of umbilical cord clamping and other strategies to influence placental transfusion at preterm birth on maternal and infant outcomes. Cochrane Database Syst Rev. 2012(8):CD003248.
17. Yao AC, Moinian M, Lind J. Distribution of blood between infant and placenta after birth. Lancet. 1969;2(7626):871-3.

18. Bhatt S, Polglase GR, Wallace EM, Te Pas AB, Hooper SB. Ventilation before Umbilical Cord Clamping Improves the Physiological Transition at Birth. Front Pediatr. 2014;2:113.
19. Knol R, Brouwer E, Vernooij ASN, Klumper F, DeKoninck P, Hooper SB, et al. Clinical aspects of incorporating cord clamping into stabilisation of preterm infants. Arch Dis Child Fetal Neonatal Ed. 2018;103(5):F493-F7.
20. Neu J. Necrotizing enterocolitis: the mystery goes on. Neonatology. 2014;106(4):289-95.
21. Bolisetty S, Dhawan A, Abdel-Latif M, Bajuk B, Stack J, Lui K, et al. Intraventricular hemorrhage and neurodevelopmental outcomes in extreme preterm infants. Pediatrics. 2014;133(1):55-62.
22. Brouwer AJ, Groenendaal F, Benders MJ, de Vries LS. Early and late complications of germinal matrix-intraventricular haemorrhage in the preterm infant: what is new? Neonatology. 2014;106(4):296-303.
23. Samuels N, van de Graaf RA, de Jonge RCJ, Reiss IKM, Vermeulen MJ. Risk factors for necrotizing enterocolitis in neonates: a systematic review of prognostic studies. BMC Pediatr. 2017;17(1):105.
24. Christensen RD, Baer VL, Del Vecchio A, Henry E. Unique risks of red blood cell transfusions in very-low-birth-weight neonates: associations between early transfusion and intraventricular hemorrhage and between late transfusion and necrotizing enterocolitis. J Matern Fetal Neonatal Med. 2013;26 Suppl 2:60-3.
25. Patel RM, Knezevic A, Shenvi N, Hinkes M, Keene S, Roback JD, et al. Association of Red Blood Cell Transfusion, Anemia, and Necrotizing Enterocolitis in Very Low-Birth-Weight Infants. JAMA. 2016;315(9):889-97.
26. Al Rifai MT, Al Tawil KI. The Neurological Outcome of Isolated PVL and Severe IVH in Preterm Infants: Is It Fair to Compare? Pediatr Neurol. 2015;53(5):427-33.
27. Al Tawil KI, El Mahdy HS, Al Rifai MT, Tamim HM, Ahmed IA, Al Saif SA. Risk factors for isolated periventricular leukomalacia. Pediatr Neurol. 2012;46(3):149-53.
28. Johnson TJ, Patel AL, Jegier BJ, Engstrom JL, Meier PP. Cost of morbidities in very low birth weight infants. J Pediatr. 2013;162(2):243-49 e1.
29. Kuint J, Lerner-Geva L, Chodick G, Boyko V, Shalev V, Reichman B, et al. Rehospitalization Through Childhood and Adolescence: Association with Neonatal Morbidities in Infants of Very Low Birth Weight. J Pediatr. 2017;188:135-41 e2.
30. Korvenranta E, Lehtonen L, Peltola M, Hakkinen U, Andersson S, Gissler M, et al. Morbidities and hospital resource use during the first 3 years of life among very preterm infants. Pediatrics. 2009;124(1):128-34.
31. Korvenranta E, Lehtonen L, Rautava L, Hakkinen U, Andersson S, Gissler M, et al. Impact of very preterm birth on health care costs at five years of age. Pediatrics. 2010;125(5):e1109-14.
32. Korvenranta E, Linna M, Rautava L, Andersson S, Gissler M, Hallman M, et al. Hospital costs and quality of life during 4 years after very preterm birth. Arch Pediatr Adolesc Med. 2010;164(7):657-63.
33. Wyllie J, Bruinenberg J, Roehr CC, Rudiger M, Trevisanuto D, Urlesberger B. European Resuscitation Council Guidelines for Resuscitation 2015: Section 7. Resuscitation and support of transition of babies at birth. Resuscitation. 2015;95:249-63.
34. de Vries LS, Eken P, Dubowitz LM. The spectrum of leukomalacia using cranial ultrasound. Behav Brain Res. 1992;49(1):1-6.
35. Volpe JJ. Intraventricular hemorrhage in the premature infant--current concepts. Part II. Ann Neurol. 1989;25(2):109-16.
36. Bell MJ, Ternberg JL, Feigin RD, Keating JP, Marshall R, Barton L, et al. Neonatal necrotizing enterocolitis. Therapeutic decisions based upon clinical staging. Ann Surg. 1978;187(1):1-7.
37. Walsh MC, Kliegman RM. Necrotizing enterocolitis: treatment based on staging criteria. Pediatr Clin North Am. 1986;33(1):179-201.

38. Pumberger W, Mayr M, Kohlhauser C, Weninger M. Spontaneous localized intestinal perforation in very-low-birth-weight infants: a distinct clinical entity different from necrotizing enterocolitis. J Am Coll Surg. 2002;195(6):796-803.
39. Samuels N, van de Graaf R, Been JV, de Jonge RC, Hanff LM, Wijnen RM, et al. Necrotising enterocolitis and mortality in preterm infants after introduction of probiotics: a quasi-experimental study. Sci Rep. 2016;6:31643.
40. Jobe AH, Bancalari E. Bronchopulmonary dysplasia. Am J Respir Crit Care Med. 2001;163(7):1723-9.
41. Walsh MC, Wilson-Costello D, Zadell A, Newman N, Fanaroff A. Safety, reliability, and validity of a physiologic definition of bronchopulmonary dysplasia. J Perinatol. 2003;23(6):451-6.
42. Hakkaart-van Roijen L, Van der Linden N, Bouwmans CAM, Kanters T, Tan SS. Costing manual: methodology of costing reaearch and reference prices for economic evaluations.; 2015.
43. M MV, K MV, S MAAE, de Wit GA, Prenger R, E AS. Dutch Tariff for the Five-Level Version of EQ-5D. Value Health. 2016;19(4):343-52.
44. Torrance GW, Furlong W, Feeny D, Boyle M. Multi-attribute preference functions. Health Utilities Index. Pharmacoeconomics. 1995;7(6):503-20.
45. Feeny D, Furlong W, Boyle M, Torrance GW. Multi-attribute health status classification systems. Health Utilities Index. Pharmacoeconomics. 1995;7(6):490-502.
46. Duley L, Dorling J, Pushpa-Rajah A, Oddie SJ, Yoxall CW, Schoonakker B, et al. Randomised trial of cord clamping and initial stabilisation at very preterm birth. Arch Dis Child Fetal Neonatal Ed. 2018;103(1):F6-F14.
47. Katheria A, Poeltler D, Durham J, Steen J, Rich W, Arnell K, et al. Neonatal Resuscitation with an Intact Cord: A Randomized Clinical Trial. J Pediatr. 2016;178:75-80 e3.
48. Winter J, Kattwinkel J, Chisholm C, Blackman A, Wilson S, Fairchild K. Ventilation of Preterm Infants during Delayed Cord Clamping (VentFirst): A Pilot Study of Feasibility and Safety. Am J Perinatol. 2017;34(2):111-6.
49. McDonald SJ, Middleton P, Dowswell T, Morris PS. Effect of timing of umbilical cord clamping of term infants on maternal and neonatal outcomes. Cochrane Database Syst Rev. 2013(7):CD004074.
50. Begley CM, Gyte GM, Devane D, McGuire W, Weeks A. Active versus expectant management for women in the third stage of labour. Cochrane Database Syst Rev. 2015(3):CD007412.
51. Richtlijn voor het uitvoeren van economische evaluaties in de gezondheidszorg. 2016.
52. Sullivan SD, Mauskopf JA, Augustovski F, Jaime Caro J, Lee KM, Minchin M, et al. Budget impact analysis-principles of good practice: report of the ISPOR 2012 Budget Impact Analysis Good Practice II Task Force. Value Health. 2014;17(1):5-14.
53. WHO. Guideline: Delayed umbilical cord clamping for improved maternal and infant health and nutrition outcomes. Geneva: World Health Organization; 2014.
